# Functional inactivation of UDP-*N*-acetylglucosamine pyrophosphorylase 1 (UAP1) induces early leaf senescence and defence responses in rice

**DOI:** 10.1093/jxb/eru456

**Published:** 2014-11-15

**Authors:** Zhaohai Wang, Ya Wang, Xiao Hong, Daoheng Hu, Caixiang Liu, Jing Yang, Yang Li, Yunqing Huang, Yuqi Feng, Hanyu Gong, Yang Li, Gen Fang, Huiru Tang, Yangsheng Li

**Affiliations:** ^1^State Key Laboratory of Hybrid Rice, College of Life Sciences, Wuhan University, Hubei 430072,China; ^2^Key Laboratory of Magnetic Resonance in Biological Systems, State Key Laboratory of Magnetic Resonance and Atomic and Molecular Physics, Wuhan Centre for Magnetic Resonance, Wuhan Institute of Physics and Mathematics, Chinese Academy of Sciences, Wuhan 430071, China; ^3^Key Laboratory of Analytical Chemistry for Biology and Medicine (Ministry of Education), Department of Chemistry, Wuhan University, Hubei, 430072, China; ^4^State Key Laboratory of Genetic Engineering, Metabolomics Laboratory, School of Life Sciences, Fudan University, Shanghai 200433, China

**Keywords:** Defence responses, leaf senescence, reactive oxygen species (ROS), rice (*Oryza sativa*), *SPL29*, UDP-*N*-acetylglucosamine pyrophosphorylase 1 (UAP1).

## Abstract

Plant leaf senescence and defence responses are important biological processes, but the molecular mechanisms involved are not well understood. This study identified a new rice mutant, *spotted leaf 29* (*spl29*). The *SPL29* gene was identified by map-based cloning, and SPL29 was confirmed as UDP-*N*-acetylglucosamine pyrophosphorylase 1 (UAP1) by enzymatic analysis. The mutant spl29 lacks UAP activity. The biological phenotypes for which UAP is responsible have not previously been reported in plants. The *spl29* mutant displayed early leaf senescence, confirmed by chlorophyll loss and photosystem II decline as physiological indicators, chloroplast degradation as a cellular characteristic, and both upregulation of senescence transcription factors and senescence-associated genes, and downregulation of photosynthesis-related genes, as molecular evidence. Defence responses were induced in the *spl29* mutant, shown by enhanced resistance to bacterial blight inoculation and upregulation of defence response genes. Reactive oxygen species, including O_2_^–^ and H_2_O_2_, accumulated in *spl29* plants; there was also increased malondialdehyde content. Enhanced superoxide dismutase activity combined with normal catalase activity in *spl29* could be responsible for H_2_O_2_ accumulation. The plant hormones jasmonic acid and abscisic acid also accumulated in *spl29* plants. ROS and plant hormones probably play important roles in early leaf senescence and defence responses in the *spl29* mutant. Based on these findings, it is suggested that UAP1 is involved in regulating leaf senescence and defence responses in rice.

## Highlight

This study identified the novel gene *UAP1* in rice. *UAP1* is involved in early leaf senescence and defence responses.

## Introduction

Leaf senescence is a complex physiological event that constitutes the last stage of leaf development, integrating multiple developmental and environmental signals (Lim and Nam, 2005; Balazadeh *et al.*, 2011). Although it has been investigated many times, the mechanisms involved are still not well understood. Early leaf senescence has been described as one of the characteristics of lesion mimic mutants (LMMs) (Qiao *et al.*, 2010; Jiao *et al.*, 2012). Leaf senescence in LMMs usually happens rapidly, providing a unique tool for dissecting possible regulatory genes or pathways during the progression of leaf growth and development to senescence. For example, *RLS1*, encoding an uncharacterized nucleotide-binding-site-containing protein, was identified and shown to be involved in autophagy-mediated chloroplast degradation during leaf senescence in rice (Jiao *et al.*, 2012). Characterization of more LMMs will help to enrich our understanding of leaf senescence.

Defence responses are important for plants in combating pathogen invasion (Dangl and Jones, 2001). Both resistance genes and many regulatory genes are involved in defence pathways. LMMs develop spontaneous lesions that resemble disease symptoms in the absence of pathogen attack, and interestingly these plants usually have a significantly enhanced resistance to disease (Jung *et al.*, 2005; Wu *et al.*, 2008). It is believed that cloning the genes responsible for LMMs is the key to deciphering the defence pathways and may aid the development of broad-spectrum pathogen resistance in plants.

UDP-*N*-acetylglucosamine pyrophosphorylase (UAP), also termed UTP:*N*-acetylglucosamine-1-P uridylyltransferase or *N*-acetylglucosamine-1-phosphate uridylyltransferase, is widely distributed in living organisms. Researchers have found that both of the human UAP1 isoforms, UAPA and UAPB (also termed AGX1 and AGX2, respectively), can use *N*-acetylglucosamine-1-phosphate (GlcNAc-1-P) or *N*-acetylgalactosamine-1-phosphate (GalNAc-1-P) as substrates for producing UDP-*N*-acetylglucosamine (UDP-GlcNAc) or UDP-*N*-acetylgalactosamine (UDP-GalNAc) (Peneff *et al.*, 2001). Enzymatic experiments with UAPs from *Arabidopsis thaliana* also showed that AtUAP1 and AtUAP2 (also termed GlcNAc1pUT-1 and GlcNAc1pUT-2, respectively) catalyse the reversible reactions (Yang *et al.*, 2010):

$$\begin{array}{l} \text{UTP}+\text{GlcNAc}-\text{1}-\text{P}/\text{GalNAc}-\text{1}-\text{P }\\ ↔\text{ UDP}-\text{GlcNAc}/\text{UDP}-\text{GalNAc}+\text{PPi} \end{array}$$

UAP is essential for organisms to grow normally. Some *UAP* mutants have been described. In *Escherichia coli*, GlmU has UAP activity, and inactivation of the *GlmU* gene rapidly reduces peptidoglycan synthesis, leading to alteration in cell shape and cell lysis (Mengin-Lecreulx and van Heijenoort, 1993). In yeast (*Saccharomyces cerevisiae*), the cells of the *ScUAP* null mutant displayed aberrant morphology in which most cells were fully swollen and some were lysed (Mio *et al.*, 1998). In *Drosophila melanogaster*, mutants of *DmUAP* (also termed *mummy*) exhibit defects in dorsal closure, central nervous system fasciculation, and eye development (Schimmelpfeng *et al.*, 2006). However, no known phenotype caused by *UAP* gene mutations has been reported in plants. In addition, the gene encoding UAP has not been identified in rice.

In order to further elucidate the molecular mechanisms of leaf senescence and defence responses in rice, a new LMM termed *spotted leaf 29* (*spl29*) was isolated. This exhibits spotted leaves and rapid leaf senescence from the seedling stage throughout the rest of its life cycle. The *SPL29* gene was identified using a map-based cloning strategy. It was confirmed that SPL29 is UAP1, with UAP activity, and that there is no enzymatic activity in the *spl29* mutant. Both early leaf senescence and defence responses were investigated and confirmed in *spl29* plants. Our data suggest that *UAP1* is probably involved in regulating leaf senescence and defence responses in rice.

## Materials and methods

### Plant material and growth conditions

The LMM mutant line *spl29* was isolated in 2008 from regenerated plants derived from a tissue culture of the rice cultivar Zhonghua 11 (ZH11, *Oryza sativa* spp. *japonica*). Its mutant phenotype, lesion mimic leaf spots and rapid leaf senescence, was stably inherited over multiple generations. M6 generation seeds of *spl29* were used in this study. Germinated wild-type and *spl29* seeds were grown in soil in a plant growth chamber (light cycle: 14h light/10h dark, 28/26°C) for use at the seedling stage, when the second-emerging leaves were used 28 days after germination. For plants used from the tillering to ripening stages, germinated wild-type and *spl29* seeds were grown in an experimental field under natural summer conditions. Leaf samples used at the tillering stage were the third leaves from the top of the main tiller of plants 50 days after germination.

### Genetic analysis and map-based cloning

For genetic analysis, the leaf phenotypes of F_1_ and F_2_ plants were observed from hybridizations of Guangzhan63s, 10N056, and Yuehui9113 with *spl29*, respectively. The F_2_ population from the cross between Guangzhan63s and *spl29* was used for preliminary and fine mapping of the *SPL29* locus. Using 44 mutant plants obtained in the F_2_ population, preliminary mapping was performed with simple sequence repeat markers, which were well distributed across all 12 chromosomes and allowed Guangzhan63s and *spl29* to be distinguished. The data for preliminary mapping were analysed using Mapmaker 3.0 software. A total of 870 F_2_ mutant plants were used for the fine mapping of *SPL29*. Nine markers (S1, S6, S8, S15, S19, S26, S32, S33, and S40) were developed for fine mapping based on DNA sequence differences between *indica* and *japonica* rice varieties. The molecular marker primer sequences are listed in Supplementary Table S1.

### Complementation of *SPL29*

The G-to-T mutation was identified by PCR amplification and sequencing using the primer pair *SPL29*Mutation (Supplementary Table S2). For complementation of the mutant phenotype, three DNA fragments of LOC_Os08g10600, the 2214-bp upstream promoter, 4674-bp gene region, and 1000-bp downstream terminator, were first amplified from the genomic DNA of wild-type Zh11 using primer pairs *SPL29*Pro, *SPL29*Gene, and *SPL29*Ter (Supplementary Table S2), respectively; and then constructed into the binary vector pBWA(V)B (reconstructed from pCAMBIA3300) using SapI sites for the digestion-link, one-step reaction. The resultant seamlessly cloned 7888-bp DNA fragment was sequenced to confirm that it was identical to the wild-type sequence in ZH11. This vector constructed for functional complementation was termed p*SPL29*C, and the empty pBWA(V)B was renamed pEmvC in this study. Vectors p*SPL29*C and pEmvC were introduced into *Agrobacterium tumefaciens* EHA105, and then transformed into calli of the *spl29* mutant. Positive transgenic plants were confirmed using *Bar178* primers (Supplementary Table S2) specific for the amplification of the *phosphinothricin* screening gene.

### Bioinformatic analysis and construction of the phylogenetic tree

The gene sequence and structure of *SPL29* were used to search the Rice Genome Annotation Project (RGAP; http://rice.plantbiology.msu.edu/). A gene family search was conducted using Pfam (http://pfam.sanger.ac.uk/). To identify the specific UDP-*N*-acetylglucosamine pyrophosphorylase encoded by *SPL29*, BLASTP searches were performed with the full-length amino acid sequence on NCBI (http://www.ncbi.nlm.nih.gov/). Sequence identities were also analysed by BLASTP.

Multiple full-length amino acid sequences were aligned using ClustalW prior to phylogenetic analysis. MEGA 5 was utilised to construct the consensus phylogenetic tree with 1000 random bootstrap replicates.

### Recombinant protein construction, expression, and purification

To generate the glutathione *S*-transferase (GST) gene fusion constructs GST-SPL29/spl29, the 1470-bp full-length coding sequence (CDS) of *SPL29* or *spl29* was amplified from the cDNA of ZH11 or *spl29* leaves, respectively (primers GST-SPL29/*spl29* CDS in Supplementary Table S2). PCR products were inserted into pGEX-6P-1 using the restriction enzyme sites BamHI and EcoRI. The recombinant vectors were transferred into *E. coli* DH5α and sequenced to check for correct construction. The empty vector pGEX-6P-1 and the recombinant vectors were then transferred into *E. coli* BL21 for protein expression. Strains harbouring *GST*, *GST-SPL29*, or *GST-spl29* were cultured in Luria-Bertani medium at 37°C. At an optical density at 600nm of 0.5, isopropylthiogalactopyranoside was added to a final concentration of 0.5mM. Strains were then cultured at 18°C for 20h (200rpm) to induce gene expression. The cells were centrifuged and resuspended in PBS buffer (137mM NaCl, 2.7mM KCl, 10mM Na_2_HPO_4_, and 2mM KH_2_PO_4_; pH 7.4). After sonication, the lysates were centrifuged. Protein purification was conducted using a column with Glutathione Sepharose^TM^ 4 Fast Flow (GE Healthcare) according to the manufacturer’s instructions. Proteins were detected by SDS-PAGE analysis and the concentration of purified protein was determined using bovine serum albumin as a standard.

### ^1^H-nuclear magnetic resonance (^1^H-NMR) analysis of SPL29/spl29 activities *in situ*

The examination of enzymatic activities was performed as described previously (Yang *et al.*, 2010) with some modifications. The forward reactions were carried out in a final volume of 540 μl, and the mixture consisted of ^2^H_2_O/H_2_O at a ratio of 8:1 (v/v), Na^+^/K^+^ phosphate buffer (80mM K_2_HPO_4_, 20mM NaH_2_PO_4_; pH 7.4), 5mM MgCl_2_, 0.2mM UTP, 0.2mM GlcNAc-1-P, 1.5 units of yeast inorganic pyrophosphatase, and recombinant enzyme (1.86 μg of GST, GST-SPL29, or GST-spl29). The reverse reactions were carried out in a similar manner, in a solution containing ^2^H_2_O/H_2_O at a ratio of 8:1, Na^+^/K^+^ phosphate buffer, 5mM MgCl_2_, 0.2mM PPi, 0.2mM UDP-GlcNAc (or UDP-GalNAc), and recombinant enzyme (1.86 μg of GST, GST-SPL29, or GST-spl29). Examination of GlcNAc-1-P/GalNAc-1-P and UDP-GlcNAc/UDP-GalNAc was performed by ^1^H-NMR as described previously (Zhang *et al.*, 2011). Data acquisition started at 10min and 60min after the addition of enzyme to the reaction mixture.

### Gene expression analysis

Samples were collected and immediately frozen in liquid nitrogen, then stored at –80°C. Total RNA was extracted using TRIzol reagent (Invitrogen). After RNase-free DNase treatment, 5 μg of RNA was used for cDNA synthesis using M-MLV reverse transcriptase (Promega) in a 50-μl reaction mixture. The quantitative, real-time reverse transcription polymerase chain reaction (qRT-PCR) technique was performed using 2×SYBR Green Master Mix reagent (Bio-Rad) in a 96-well plate using a Bio-Rad CFX96 real-time PCR system. Three technological replicates were used for each biological sample. Six rice reference genes (from our unpublished data on the selection of stable reference genes to normalise gene expression in rice) were assessed for their potential as stable internal standards by geNorm as previously described (Vandesompele *et al.*, 2002). These genes were *TI* (LOC_Os01g05490), *ARF* (LOC_Os05g41060), *EF-1α* (LOC_Os03g08020), *UBC* (LOC_Os02g42314), *Profilin-2* (LOC_Os06g05880), and *Actin1* (LOC_Os03g50885). As a result of this analysis, *UBC*, *Profilin-2*, and *Actin1* were selected as internal standards for all leaf samples (Supplementary Figure S1). All primers used for qRT-PCR analysis are listed in Supplementary Table S3, with good PCR efficiencies (85–105%) assessed using a 10-fold dilution series of total cDNA.

### Chlorophyll content and chlorophyll *a* fluorescence

Chlorophyll was extracted from leaves with ice-cold 80% acetone, and the chlorophyll content per gram of leaf fresh weight (FW) was determined as previously described (Lichtenthaler, 1987).

Chlorophyll *a* fluorescence transients from dark-adapted plants were measured using a Handy Plant Efficiency Analyser (Handy PEA, Hansatech, UK). The description and calculation formulae of parameters are listed in Supplementary Table S4.

### Transmission electron microscopy

Leaf segments were fixed with 2.5% glutaraldehyde in sodium phosphate buffer (pH 7.2) for 4h at 4°C. Ultrathin samples were made as previously described (Zhou *et al.*, 2013), and viewed using a transmission electron microscope.

### Inoculations with bacterial blight pathogen

*Xanthomonas oryzae* pv. *oryzae* strain PXO99 (Philippine race 6) was cultured on pressure-sensitive adhesive medium. The second and third youngest fully expanded leaves from both the main and lateral tillers of 5–7 independent wild-type and *spl29* plants were inoculated with PXO99 suspensions (optical density of 0.5 at 600nm) using the leaf clipping method (Manosalva *et al.*, 2011). Lesion lengths on inoculated plants were measured 12 days after inoculation.

### Histochemical staining

Leaves in wild-type and *spl29* plants were sampled at the tillering stage (about 45 days after germination under natural summer field conditions). Histochemical assays for determining reactive oxygen species (ROS) accumulation were conducted as previously described (Qiao *et al.*, 2010). Three independent experiments were performed.

### Lipid peroxidation and ROS-scavenging enzyme assays

Plant samples were ground in liquid nitrogen. Crude protein extraction, measurement of malondialdehyde (MDA) contents, and assays for the activities of superoxide dismutase (SOD) and catalase (CAT) were conducted according to previously described methods (Wang *et al.*, 2012).

### Quantification of jasmonic acid and abscisic acid

Plant leaves were collected, weighed, immediately frozen in liquid nitrogen, and stored at –80°C. Extraction and measurement of jasmonic acid (JA) and abscisic acid (ABA) were conducted following a previously described method (Chen *et al.*, 2012).

## Results

### Characterization of the *spl29* mutant

**Fig. 1.**
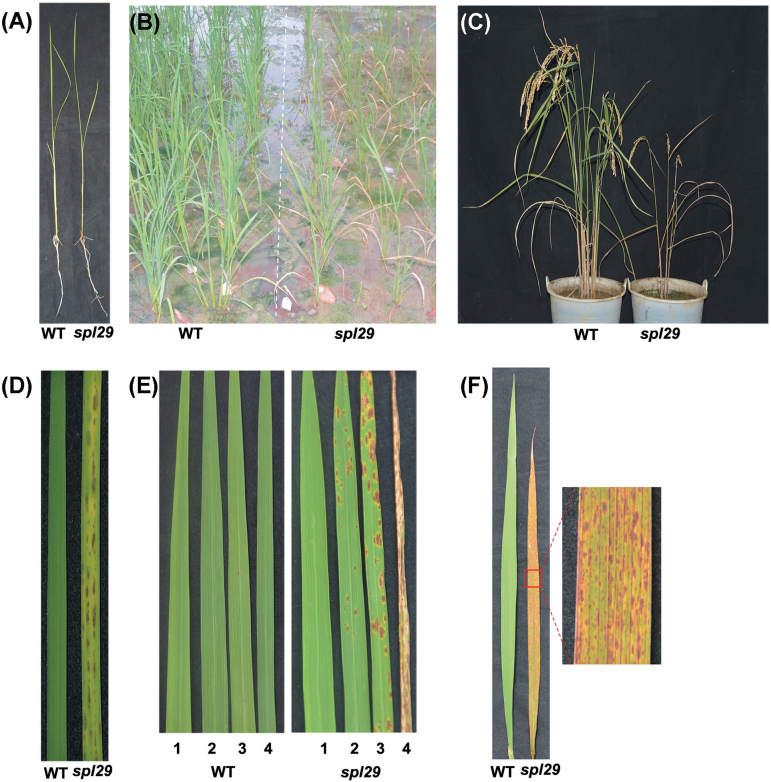
Leaf phenotype of wild-type and *spl29* mutant plants. (A) Plants at the seedling stage (28 days after germination). (B) Plants at the tillering stage (50 days after germination). (C) Plants at the ripening stage (115 days after germination). (D) Phenotype of the second-emerged leaf after germination of plants at the seedling stage, corresponding to (A). (E) Phenotype of the first, second, third, and fourth fully expanded leaves from the tip to the base of the main tiller of plants at the tillering stage, corresponding to (B). (F) Flag-leaf phenotype of plants 90 days after germination. The *spl29* leaf area in the red square frame is magnified on the right.

Small, dark-brown LM leaf spots (followed by rapid leaf senescence) appeared on *spl29* plants from the seedling stage to the ripening stage (Fig. 1A, B, and C). However, newly emerging leaves showed no difference to wild-type plants. During the seedling stage, abundant leaf spots had appeared on *spl29* plants by 28 days after germination, with leaves exhibiting chlorosis beside the spots (Fig. 1D). After the appearance of leaf spots and chlorosis, the leaves withered from the tip to the base in about five days (Supplementary Figure S2). The severity of leaf mutant phenotypes varied for different leaves of a single *spl29* plant at the tillering stage 50 days after germination (Fig. 1E). No spots were present on the first new fully expanded leaf, whereas the third leaf from the top of the main tiller had larger and more numerous spots and associated chlorosis than the second leaf, and at this point the fourth leaf was already withered and dead. By contrast, the corresponding leaves on the wild-type plant grew normally. When the last emerging flag leaf was filled with spots (Fig. 1F), the *spl29* plants began to senesce far earlier than wild-type plants. In addition to the obvious leaf phenotype, the plant height, panicle length, total grain number, filled grain number, seed-setting rate, and thousand-grain weight of the *spl29* mutants were all significantly decreased when compared with the wild-type plants (Table 1).

**Table 1.** Agronomic traits of wild-type and *spl29* plants^a^

	Plant height (cm)	Panicle length (cm)	Total grain number	Filled grain number	Seed-setting rate (%)	Thousand-grain weight (g)
WT	89.9±2.5	19.2±0.8	942±116	871±104	92.5±2.3	24.59±0.32
*spl29*	68.8±3.5***	15.3±0.9***	601±113***	249±44***	41.4±1.2***	21.97±0.22***

^a^ Data represent the mean ± SD of nine (WT) or seven (*spl29*) independent plants (Student’s *t*-test, ***, *P* < 0.0005). WT, wild type.

### Map-based cloning of the *SPL29* gene

For genetic analysis, three *indica* cultivars, Guangzhan63s, 10N056, and Yuehui9113, were selected for hybridisation with *spl29*. All F_1_ plants displayed the wild-type leaf phenotype. In all three F_2_ populations, the wild-type and LM mutant phenotypes segregated at a ratio of 3:1 (χ^2^ < χ^2^_0.05_ = 3.84; *P* > 0.05; see Supplementary Table S5), which suggested that the phenotype of *spl29* was controlled by a single recessive nuclear gene.

**Fig. 2.**
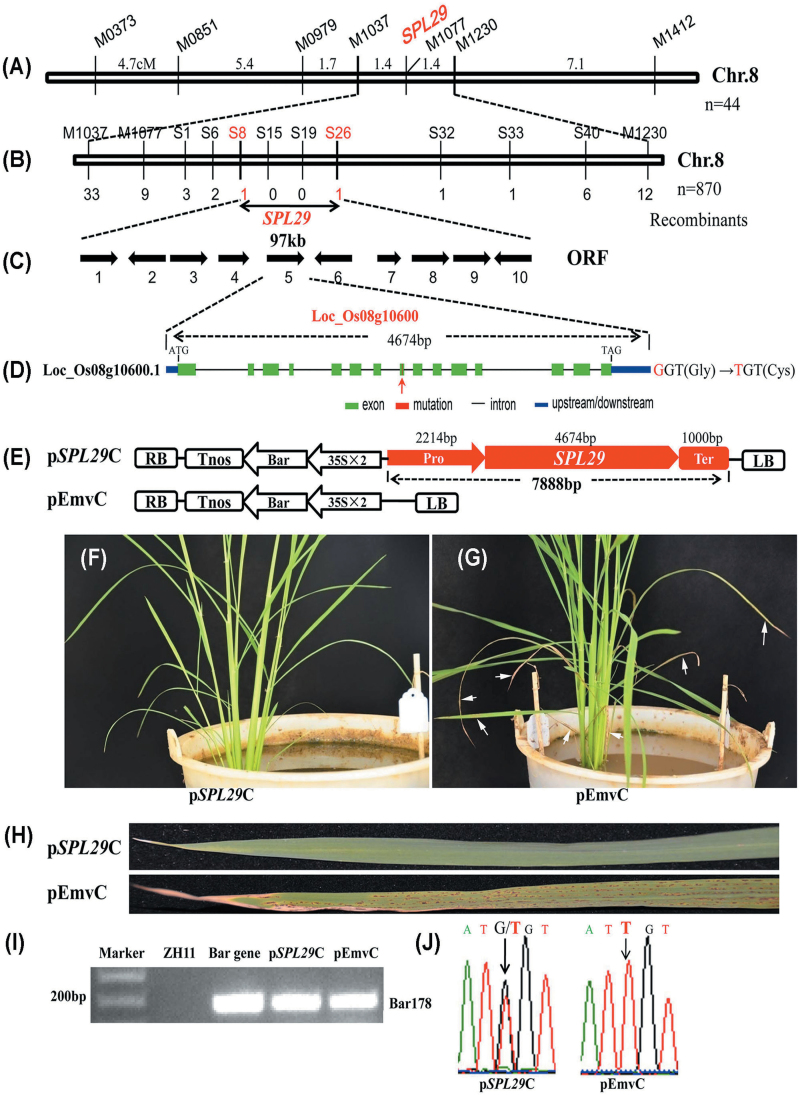
Map-based cloning and functional complementation of *SPL29*. (A) Preliminary mapping of the *SPL29* locus. (B) Fine mapping of the *SPL29* locus. (C) Ten putative ORFs are located in the 97-kb region identified by fine mapping. (D) Gene structure of the *SPL29* candidate LOC_Os08g10600. Exons, introns, and upstream/downstream regions are displayed. The point mutation of G to T on the eighth exon is indicated by the red arrow, leading to the amino acid exchange of Gly to Cys. (E) Schematic diagram of vectors for functional complementation. The p*SPL29*C functional complementation vector contains the promoter, gene region, and terminator of LOC_Os08g10600; pEmvC is the empty vector control. The p*SPL29*C and pEmvC vectors were transformed into *spl29* calli. LB, left border; RB, right border; 35S, cauliflower mosaic virus 35S promoter; Bar, the *phosphinothricin* gene; Tnos, the *nopaline synthase* terminator. (F–G) Transgenic plants of p*SPL29*C and pEmvC about 40 days after regeneration. Arrows indicate leaves with the mutant phenotype. (H) Clear leaf phenotype in transgenic plants of p*SPL29*C and pEmvC. (I) Positive amplification of the transgenic marker element (*Bar* gene) in transgenic plants of p*SPL29*C and pEmvC. ZH11 DNA was used as the negative control and the plasmid pEmvC was used as the positive control. Bar178, amplicon of the *Bar* gene with a length of 178bp. (J) Sequence analysis of the G-to-T mutation site in transgenic plants of p*SPL29*C and pEmvC.

The F_2_ population from the cross between Guangzhan63s and *spl29* was used to map the *SPL29* gene. Using 44 F_2_ mutant plants, the *SPL29* gene was closely linked with marker M1077, and mapped inside markers M1037 and M1230 on chromosome 8, with an equal genetic distance of 1.4 cM to each (Fig. 2A). For fine mapping of the *SPL29* gene, more than 3000 F_2_ individuals were generated and new markers between M1037 and M1230 were designed according to sequence differences between *indica* and *japonica* rice. Nine markers showing polymorphisms between Guangzhan63s and *spl29* (Supplementary Table S1) were used to screen the recombinants. Using 870 F_2_ mutant plants, the *SPL29* gene was eventually limited to a 97-kb region between the new markers S8 and S26, with one recombinant for each marker (Fig. 2B). Ten putative open reading frames (ORFs) were predicted according to the RGAP website (Fig. 2C). All ten genes were amplified and sequenced. By comparing gene sequences between wild-type and *spl29* plants, one point mutation was identified on the fifth gene, LOC_Os08g10600. No DNA sequence changes were found in the other nine genes or the putative promoter region (about 2.2kb) of LOC_Os08g10600. The single nucleotide substitution of guanine (G) to thymine (T) occurred in the eighth exon of LOC_Os08g10600 (at a position of 712bp in the CDS), resulting in a single amino acid change from glycine (Gly) to cysteine (Cys) (Fig. 2D).

For preliminary confirmation that the G-to-T mutation was responsible for the phenotype of the *spl29* mutant, this nucleotide site was examined in different rice cultivars, F_2_ non-lesion-mimic (NLM) plants, and F_2_ LM plants (Supplementary Table S6). The nucleotide at this site was T for *spl29* and all tested F_2_ LM plants, but G for 10 different normal rice cultivars. Four of the F_2_ NLM plants had a G at this position, while G and T existed together at this site in seven of the F_2_ NLM plants. Thus, LOC_Os08g10600 was a likely *SPL29* candidate.

### Functional complementation with LOC_Os08g10600 in the *spl29* mutant

A 7888-bp genomic fragment of wild-type Zh11, containing the 2214-bp upstream promoter, the 4674-bp gene region, and the 1000-bp downstream terminator of LOC_Os08g10600, was constructed using the plasmid p*SPL29*C and transferred into the *spl29* calli by *Agrobacterium tumefaciens*-mediated transformation. The empty vector pEmvC was introduced to *spl29* calli as the control (Fig. 2E). About 40 days after plant regeneration, all 22 independent transgenic lines containing the wild-type *SPL29* gene showed a complete rescue of the mutant phenotype (Fig. 2F), while all 16 independent transgenic lines with the empty vector failed to complement the *spl29* mutant (Fig. 2G). Leaf phenotypes of the transgenic plants with p*SPL29*C or pEmvC are shown in Fig. 2H. Successfully transformed plants were confirmed by PCR amplification of the screening marker *Bar* gene (Fig. 2I). Sequencing the G-to-T mutation site of the *SPL29* gene revealed that plants transformed with p*SPL29C* had both G and T at the mutant nucleotide site, whereas the pEmvC control plants only had the mutant T nucleotide (Fig. 2J). Therefore, functional complementation with the wild-type candidate gene rescued the mutant phenotype of *spl29* plants, and it was concluded that LOC_Os08g10600 was the *SPL29* gene.

### *SPL29* encodes a putative UAP1

**Fig. 3.**
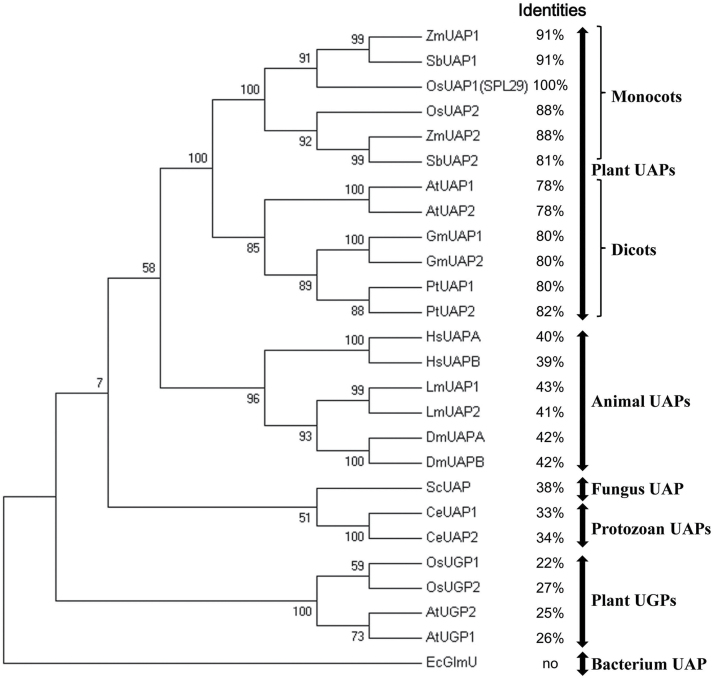
Phylogenetic analysis of UAPs in different organisms. The tree was constructed based on the full-length amino acid sequences of UAPs and UGPs. ZmUAP1, *Zea mays* (AFW56989.1); SbUAP1, *Sorghum bicolour* (XP_002444024.1); OsUAP1(SPL29), *Oryza sativa* (NP_001061242.1); OsUAP2, *O. sativa* (NP_001053857.1); ZmUAP2, *Z. mays* (NP_001266496.1); SbUAP2, *S. bicolour* (XP_002448519.1); AtUAP1, *Arabidopsis thaliana* (NP_564372.3); AtUAP2, *A. thaliana* (NP_181047.1); GmUAP1, *Glycine max* (XP_003524811.1); GmUAP2, *G. max* (XP_003531103.1); PtUAP1, *Populus trichocarpa* (XP_002303345.2); PtUAP2, *P. trichocarpa* (XP_006369046.1); HsUAPA, *Homo sapiens* (NP_003106.3, isoform A); HsUAPB, *H. sapiens* (Q16222.3, isoform B); LmUAP1, *Locusta migratoria* (JX484802.1); LmUAP2, *L. migratoria* (JX484803.1); DmUAPA, *Drosophila melanogaster* (NP_609032.1, isoform A); DmUAPB, *D. melanogaster* (NP_723183.1, isoform B); ScUAP, *Saccharomyces cerevisiae* (NP_010180.1); CeUAP1, *Caenorhabditis elegans* (NP_497777.1); CeUAP2, *C. elegans* (NP_500511.2); OsUGP1, *O. sativa* (NP_001063879.1); OsUGP2, *O. sativa* (NP_001045689.1); AtUGP2, *A. thaliana* (NP_197233.1); AtUGP1, *A. thaliana* (NP_186975.1); EcGlmU, *Escherichia coli* (P0ACC7.1). Sequence identities with SPL29 are indicated for different UAPs and UGPs, and ‘no’ indicates no sequence identity.

As annotated on RGAP, LOC_Os08g10600 (*SPL29*) encodes a putative 489-amino acid protein. Pfam analysis with its predicted amino acid sequence showed that *SPL29* belongs to the UDP-glucose pyrophosphorylase gene family (PF01704). NCBI BLASTP revealed that *SPL29* is a putative *UAP1* gene in rice (*OsUAP1*), but not the *UDP-glucose pyrophosphorylase* (*UGP*) gene. In the rice genome, one homologue of *SPL29* is also annotated as *UAP* (LOC_Os04g52370, termed *OsUAP2*). To examine the evolutionary relationships between SPL29 and its homologues in different species, a bootstrap consensus phylogenetic tree was constructed by the neighbour-joining method (Fig. 3). All UAPs in different eukaryotic organisms clustered separately from the UGPs. The protein EcGlmU, which shows UAP activity in *E. coli*, showed no amino acid homology with SPL29, and was thus located furthest away. UAPs in plants were tightly clustered, with separate groups for monocots and dicots, indicating that gene divergence occurred after diversification of these two clades. In addition, SPL29 showed over 30% amino acid identity with all eukaryotic UAPs analysed by NCBI BLASTP (Fig. 3). These results all suggest that *SPL29* encodes a putative UAP1 in rice.

### SPL29 shows UAP enzymatic activities but spl29 eliminates these enzymatic functions

UAP has not been identified in rice before. In order to reveal whether SPL29 and the mutant spl29 have UAP activity, recombinant proteins of GST-SPL29 and GST-spl29 were produced. Theoretically, the molecular weights are 26kDa for GST, 54.071kDa for SPL29, and 54.117kDa for spl29. GST, GST-SPL29 (about 80kDa), and GST-spl29 were highly expressed after induction (Supplementary Figure S3, lanes 2–4). Column-purified proteins were used to detect the enzymatic reactions (Supplementary Figure S3, lanes 5–7).

**Fig. 4.**
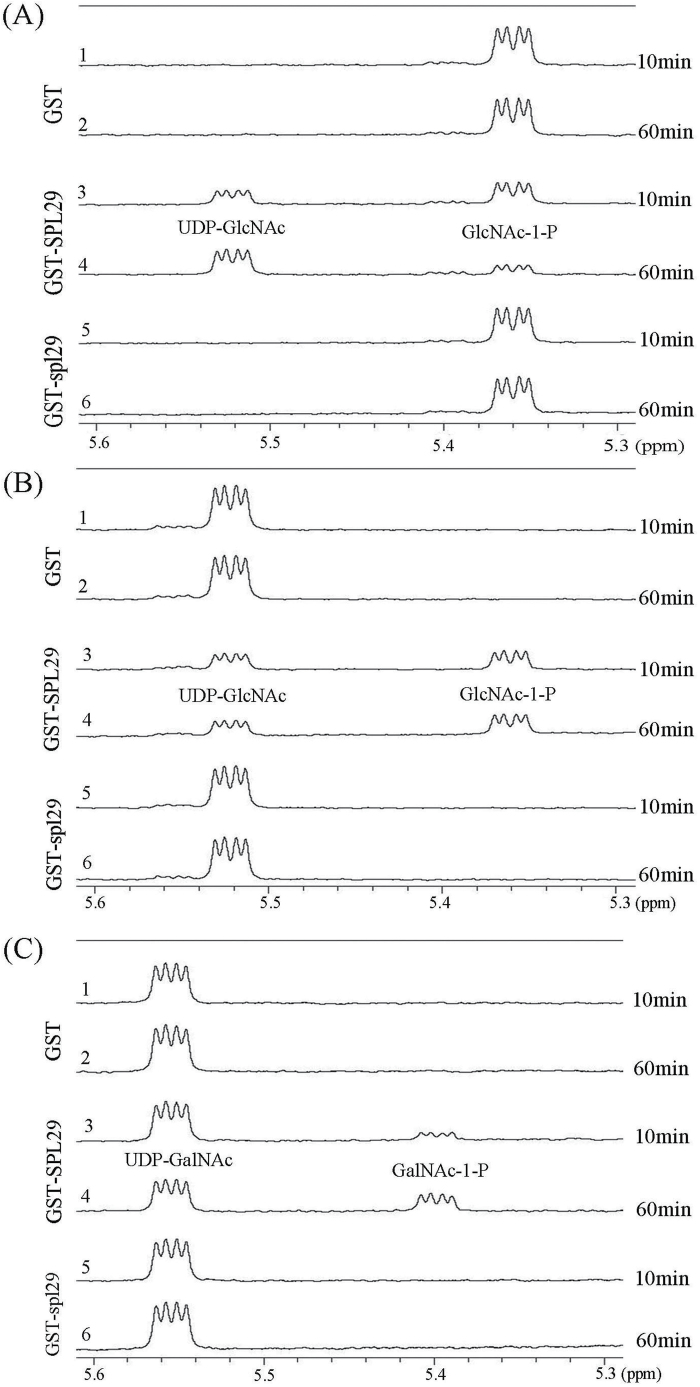
Enzymatic activities of SPL29 and spl29 based on ^1^H-NMR. (A) Forward activity:UTP+GlcNAc-1-P→UDP-GlcNAc+PPi The chemical shift (indicated by the ‘peak’ shape) is 5.36 ppm for the substrate GlcNAc-1-P and 5.52 ppm for the product UDP-GlcNAc. (B) Reverse activity:UDP-GlcNAc+PPi→GlcNAc-1-P+UTP The chemical shift is 5.52 ppm for the substrate UDP-GlcNAc and 5.36 ppm for the product GlcNAc-1-P. (C) Reverse activity:UDP-GalNAc+PPi→GalNAc-1-P+UTP The chemical shift is 5.55 ppm for the substrate UDP-GalNAc and 5.39 ppm for the product GalNAc-1-P. In (A), (B), and (C), data acquisition started at 10min and 60min after the addition of the protein (GST, GST-SPL29, or GSP-spl29) to the reaction mixture. The line number indicates each measurement. Results are representative of two independent experiments.

^1^H-NMR spectroscopy was used to monitor the enzymatic reaction of SPL29 and spl29 *in situ*. In the time-gradient enzymatic progression at 10min and 60min, forward conversion of GlcNAc-1-P (5.36 ppm) to UDP-GlcNAc (5.52 ppm) was observed with GST-SPL29 (Fig. 4A, lines 3 and 4), but not with the GST control (Fig. 4A, lines 1 and 2). Interestingly, GST-spl29 was unable to catalyse this reaction (Fig. 4A, lines 5 and 6). Similarly, reverse conversion of UDP-GlcNAc (5.52 ppm) to GlcNAc-1-P (5.36 ppm) was observed with GST-SPL29 (Fig. 4B, lines 3 and 4), but not with GST (Fig. 4B, lines 1 and 2) or GST-spl29 (Fig. 4B, line 5 and 6). GST-SPL29 could also catalyse the reverse conversion of UDP-GalNAc (5.55 ppm) to GalNAc-1-P (5.39 ppm) (Fig. 4C, lines 3 and 4), whereas GST (Fig. 4C, lines 1 and 2) and GST-spl29 (Fig. 4C, lines 5 and 6) could not. The forward reaction for GalNAc-1-P could not be tested since GalNAc-1-P was commercially unavailable.

These NMR-based assays provide unambiguous evidence that SPL29 has UAP activity whereas the mutation of SPL29 to spl29 eliminates this enzymatic function. To date, UAP has not been well studied in plants. The *spl29* mutant can be used to reveal the biological significance of UAP.

### Early leaf senescence in the *spl29* mutant is identified by physiological indicators

**Fig. 5.**
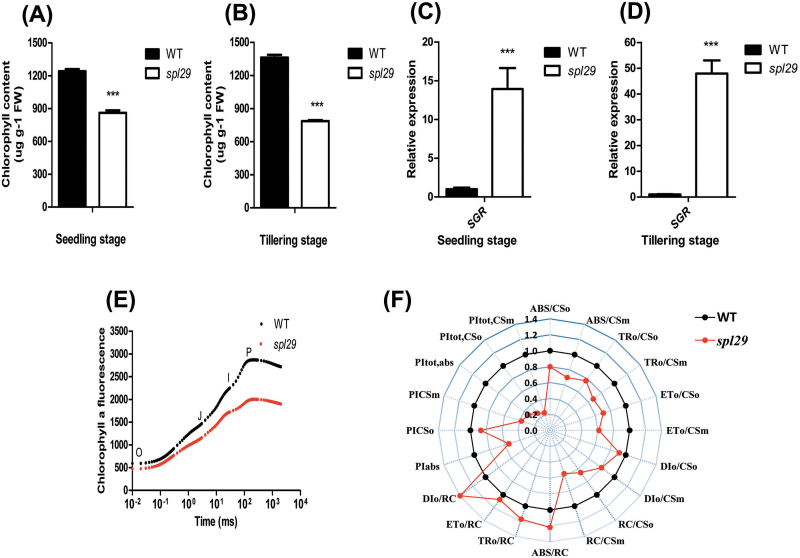
Identification of leaf senescence in *spl29* at the physiological level. (A, B) Chlorophyll contents. FW, fresh weight. (C, D) Relative expression of the senescence-induced *SGR* gene by qRT-PCR. The expression level of *SGR* in the wild type was normalised to 1. In A–D, wild-type and *spl29* leaf samples were analysed at the seedling and tillering stages; data represent the mean ± SD of three or four biological replicates (Student’s *t*-test: ***, *P* < 0.0005). (E) Chlorophyll *a* fluorescence transients. (F) Analysis of fluorescence parameters (see also Supplementary Table S4). (E–F) Each data point is the mean value of ten independent plants.

The decrease of chlorophyll content is commonly used as a physiological indicator related to plant leaf senescence (Jiao *et al.*, 2012). At the seedling stage, chlorophyll content decreased from 1240 μg g^–l^ FW in wild-type leaves to 861 μg g^–1^ FW in *spl29* leaves (Fig. 5A). A similar reduction was also observed at the tillering stage, when chlorophyll contents were 1362 μg g^–1^ FW in the wild type and 786 μg g^–1^ FW in *spl29* (Fig. 5B). The senescence-induced *STAY GREEN* (*SGR*) gene plays an important role in regulating chlorophyll degradation (Park *et al.*, 2007). Analysis by qRT-PCR showed that the expression of *SGR* was greatly upregulated in *spl29* leaves at the seedling and tillering stages (Fig. 5C–D).

Photosystem II (PSII) function is usually reduced during senescence. The chlorophyll *a* fluorescence transient can be examined to reveal the activities of PSII machinery on light absorption, energy transformation, and electron transfer, thus this technique is commonly used to monitor leaf senescence (Zhang *et al.*, 2012; Panda and Sarkar, 2013). Wild-type and *spl29* plants at the seedling stage were analysed. In both lines, chlorophyll *a* fluorescence transients showed a typical polyphasic rise with the basic steps O-J-I-P, but the fluorescence intensity was reduced in *spl29* plants (Fig. 5E). Greater amplitude of ΔW, increased K (300 μs), J (2ms), and I (30ms) steps, positive ΔL_band_, and the maximum amplitude of the IP phase implied PSII reduction (Supplementary Figure S4). Data analysis of chlorophyll *a* fluorescence transients is shown in Fig. 5F. Absorption flux (ABS/CS_o_ and ABS/CS_m_), trapped energy flux (TR_o_/CS_o_ and TR_o_/CS_m_), electron transport flux (ET_o_/CS_o_ and ET_o_/CS_m_), and dissipated energy flux (DI_o_/CS_o_ and DI_o_/CS_m_) per excited cross section (CS) were all decreased in *spl29* plants, indicating the destruction of the entire PSII machinery. Interestingly, although the density of reaction centres (RCs) (RC/CS_o_ and RC/CS_m_) decreased, the capacities per RC (ABS/RC, TR_o_/RC, ET_o_/RC, and DI_o_/RC) increased in *spl29* plants. However, increased capacities per RC did not rescue the capacity of the PSII machinery in *spl29*, which was completely reduced as clearly reflected by performance indices (PI_ABS_, PI_CSo_, PI_CSm_, PI_tol,ABS_, PI_tol,CSo_, and PI_tol,CSm_). These results demonstrated that the PSII units were damaged and could not function properly in *spl29* leaves.

The decreased chlorophyll content and reduced PSII capacity indicated that the *spl29* plants had entered senescence at the physiological level.

### Irreversible degradation of chloroplasts in early-senescent leaves of *spl29* mutants

**Fig. 6.**
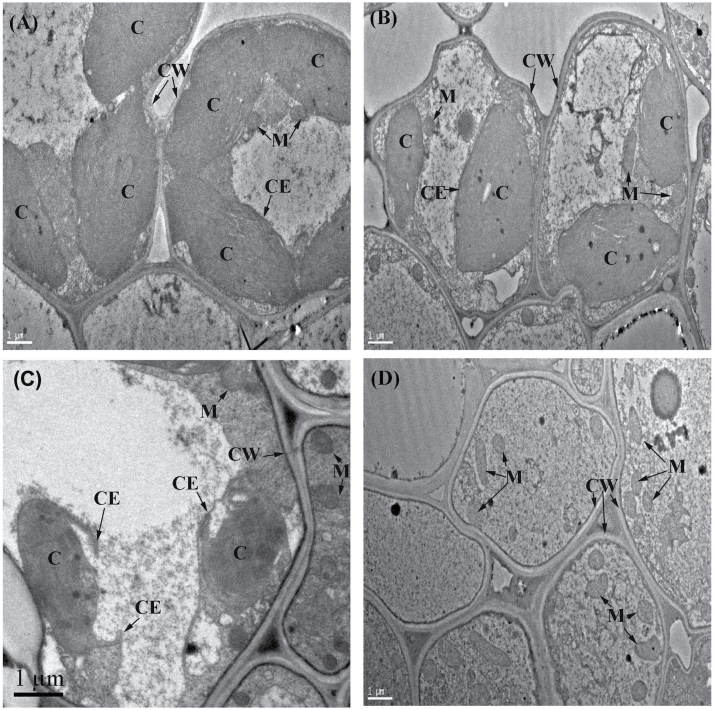
Identification of leaf senescence in *spl29* at the cellular level by ultrastructural analysis. (A) Well developed wild-type mesophyll cells. (B) Well developed *spl29* mesophyll cells. (C) Chloroplast envelope in *spl29* breaking. (D) Chloroplasts completely degraded in mesophyll cells of *spl29*. C, chloroplast; M, mitochondrion; CW, cell wall; CE, chloroplast envelope. All scale bars represent 1 μm.

The main cellular characteristic of leaf senescence is the occurrence of chloroplast degradation (Lim *et al.*, 2007; Jiao *et al.*, 2012). To investigate whether the chloroplasts were affected in the early-senescing leaf of the *spl29* mutant, the ultrastructures of wild-type and *spl29* cells in 28-day-old seedling leaves were compared using transmission electron microscopy. In wild-type leaves, well-developed mesophyll cells were observed with fully developed and membrane-intact chloroplasts (Fig. 6A). However, three different chloroplast states were observed among *spl29* mesophyll cells. Fully developed and membrane-intact chloroplasts could be observed in many *spl29* mesophyll cells (Fig. 6B), while in some cells the chloroplast envelope was observed to be breaking (Fig. 6C and Supplementary Figure S5A and B). Unsurprisingly, mesophyll cells which had lost chloroplasts but retained mitochondria were present abundantly in *spl29* (Fig. 6D and Supplementary Figure S5C and D). These results suggest that chloroplasts developed normally in *spl29* at first, but were completely degraded during early leaf senescence. Chloroplast breakage may be one mechanism causing this degradation. Drastic, irreversible, and complete degradation of chloroplasts in *spl29* leaves is coincident with the phenotype of rapid early leaf senescence (leading to death).

### Upregulated expression levels of senescence-associated transcription factors and senescence-associated genes, and downregulated expression levels of photosynthesis-related genes, are molecular evidence of early leaf senescence in the *spl29* mutant

**Fig. 7.**
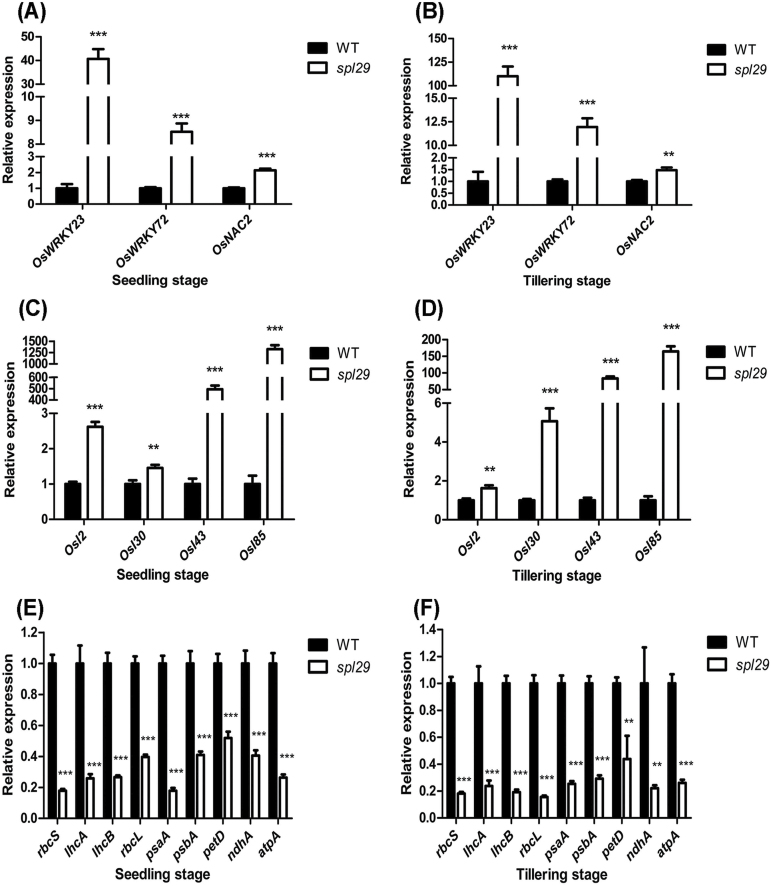
Identification of leaf senescence in *spl29* at the molecular level by qRT-PCR. (A, B) Relative expression of senescence-associated transcription factors. (C, D) Relative expression of *SAG*s. (E, F) Relative expression of photosynthesis-related genes. Wild-type and *spl29* leaf samples were analysed at the seedling and tillering stages; the expression level of each gene in the wild type was normalised to 1. Data represent the mean ± SD of three biological replicates (Student’s *t*-test: **, *P* < 0.005; ***, *P* < 0.0005).

Leaf senescence is a complex process controlled by a large number of different genes. Many genes that encode transcription factors are upregulated to induce leaf senescence (Zhou *et al.*, 2013). To further confirm that senescence occurred in the *spl29* plants, gene expression analysis of three senescence-associated transcription factors (*OsWRKY23*, *OsWRKY72*, and *OsNAC2*) was performed by qRT-PCR. At the seedling stage, transcripts of *OsWRKY23*, *OsWRKY72*, and *OsNAC2* in *spl29* leaves increased 40.7-, 8.5-, and 2.1-fold, respectively, in comparison to those of wild-type leaves (Fig. 7A); at the tillering stage their levels in *spl29* leaves were increased 110.0-, 12.0-, and 1.5-fold, respectively, compared to the wild type (Fig. 7B). The expression levels of the senescence-associated transcription factors investigated here were all upregulated, according with the early leaf senescence of the *spl29* mutant.

Senescence-associated genes (SAGs) are upregulated during senescence and thought to be involved in triggering the process or controlling its rate of progression (Lee *et al.*, 2001). Expression levels of four SAGs (*Osl2*, *Osl30*, *Osl43* and *Osl85*) were determined by qRT-PCR. At the seedling stage, in *spl29* leaves, *Osl2*, *Osl30*, *Osl43*, and *Osl85* mRNAs were 2.6-, 1.5-, 494.4-, and 1324.4-fold higher, respectively, than they were in wild-type leaves (Fig. 7C); at the tillering stage their levels in *spl29* leaves were 1.6-, 5.1-, 83.5- and 164.7-fold more abundant, respectively, than those in wild-type leaves (Fig. 7D). The upregulated expression patterns of SAGs further support the notion that early leaf senescence occurred in *spl29* plants.

Leaf senescence is accompanied by the decreased expression of genes related to photosynthesis (Lim *et al.*, 2007). The expression levels of three nuclear encoded genes (*rbcS*, *lhcA*, and *lhcB*) and six chloroplast-encoded genes (*rbcL*, *psaA*, *psbA*, *petD*, *ndhA*, and *atpA*) associated with photosynthesis were assessed by qRT-PCR. At the seedling stage, expression levels of *rbcS*, *lhcA*, *lhcB*, *rbcL*, *psaA*, *psbA*, *petD*, *ndhA*, and *atpA* in *spl29* were 0.18-, 0.26-, 0.27-, 0.40-, 0.18-, 0.41-, 0.52-, 0.41-, and 0.26-fold, respectively, of those in the wild type (Fig. 7E). At the tillering stage, expression levels of these nine genes in *spl29* were 0.18-, 0.24-, 0.19-, 0.16-, 0.25-, 0.29-, 0.44-, 0.22-, and 0.26-fold, respectively, as much as those in the wild type (Fig. 7F). Downregulation of these photosynthesis-associated genes also provided molecular evidence for early leaf senescence in *spl29* plants.

### Plant resistance to the bacterial blight pathogen is enhanced and expression levels of defence signalling-related genes are induced in the *spl29* mutant

**Fig. 8.**
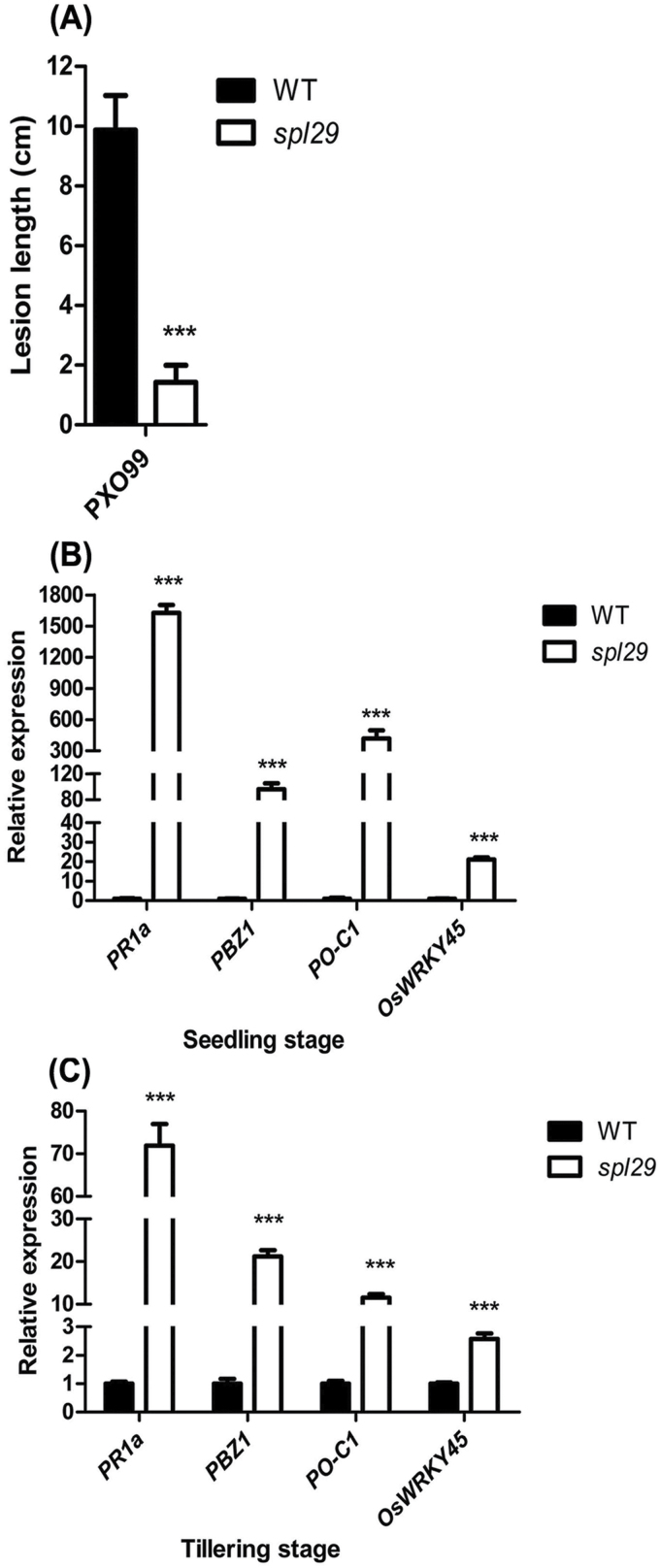
Detection of disease resistance and expression of defence signalling-related genes in wild-type and *spl29* plants. (A) Mean lesion length after inoculation of plant leaves with bacterial blight pathogen PXO99. Data represent the mean ± SD from 5–7 independent plants at the tillering stage (Student’s *t*-test: ***, *P* < 0.0005). (B, C) Relative expression of four defence signalling-related genes in wild-type and *spl29* plants at the seedling and tillering stages analysed by qRT-PCR. The expression level of each gene in the wild type was normalised to 1; data represent the mean ± SD of three biological replicates (Student’s *t*-test: ***, *P* < 0.0005).

The appearance of LM spots in *spl29* plants resembles the hypersensitive response (HR), an important resistance mechanism in plants (Dangl and Jones, 2001; Lam *et al.*, 2001). To test their resistance to pathogens, *spl29* and wild-type plants were inoculated with the bacterial blight strain PXO99 at the tillering stage. The wild-type plants showed a typical response to bacterial blight disease, while the *spl29* plants exhibited significantly enhanced resistance. The average leaf lesion lengths following PXO99 inoculation were 9.87cm in wild-type plants and 1.42cm in *spl29* plants (Fig. 8A).

Activation of defence-response gene expression has previously been observed during the development of lesions in some rice LMMs (Yin *et al.*, 2000; Manosalva *et al.*, 2011). Therefore, we examined the expression of four defence signalling-related genes (*PR1a*, *PBZ1*, *PO-C1*, and *OsWRKY45*) in wild-type and *spl29* plants by qRT-PCR. At the seedling stage, levels of *PR1a*, *PBZ1*, *PO-C1*, and *OsWRKY45* mRNAs in the *spl29* mutant were 1627.5-, 96.2-, 419.5-, and 21.1-fold, respectively, the amount of those in wild-type plants (Fig. 8B); at the tillering stage these genes were expressed 71.9-, 21.2-, 11.6-, and 2.6-fold, respectively, as much in *spl29* as they were in wild-type plants (Fig. 8C). The upregulation of defence signalling-related genes indicates that the plant defence responses are induced, which may contribute to enhancing disease resistance in the *spl29* mutant.

### ROS and MDA accumulate in the *spl29* mutant accompanied by increased SOD activity and normal CAT activity

**Fig. 9.**
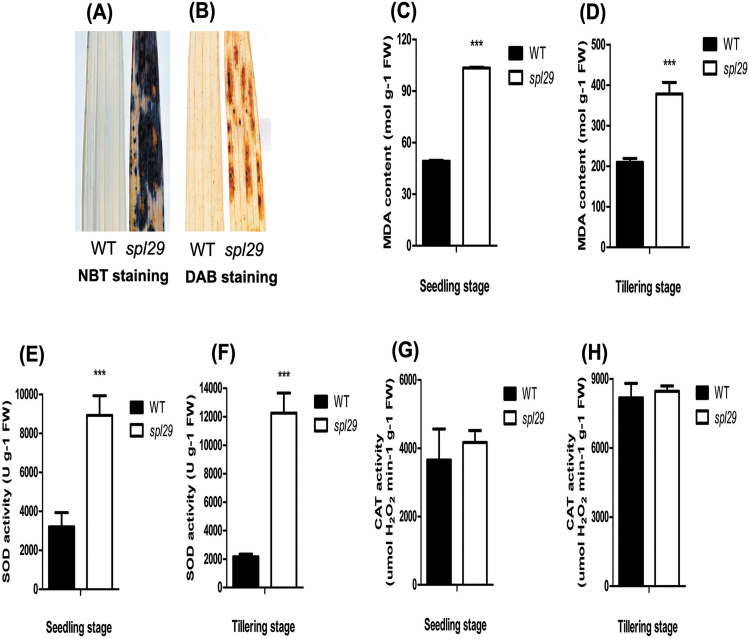
ROS accumulation in wild-type and *spl29* leaves. (A-B) Histochemical detection of O_2_^–^ by NBT staining and H_2_O_2_ by DAB staining: purple formazan precipitate indicates the location of O_2_^–^ and brown precipitate indicates the location of H_2_O_2_. Plant leaves at the tillering stage were analysed; results are representative of three independent experiments. (C, D) MDA contents. (E, F) SOD activities. U: 1U = 1 SOD activity unit (inhibition of 50%). (G, H) CAT activities. For C–H, leaf samples were analysed at the seedling and tillering stages; data represent the mean ± SD of three biological replicates (Student’s *t*-test: ***, *P* < 0.0005). FW, fresh weight.

ROS generation is one of the earliest responses of plant cells during senescence (Khanna-Chopra, 2012). ROS also play important roles in plant–pathogen interactions (Wojtaszek, 1997; Apel and Hirt, 2004). This prompted us to investigate whether ROS accumulation occurred in *spl29* plants. Leaves at the tillering stage were used for the ROS assay. The pattern of nitro blue tetrazolium (NBT) staining reflected the formation of blue formazan precipitates and was indicative of O_2_^–^ accumulation. There was extensive NBT staining in the leaf area surrounding lesions in *spl29* plants, whereas staining was minimal in wild-type leaves (Fig. 9A). When 3,3’-diaminobenzidine (DAB) staining was used as an indicator of H_2_O_2_ accumulation, intense brown staining was seen correlating with lesion formation in *spl29* mutant leaves, but no such signal was detected in wild-type leaves (Fig. 9B). These results demonstrated that ROS accumulated in *spl29* plants.

Lipid peroxidation by ROS was analysed by measuring contents of MDA, an end-product of oxidized lipids. At the seedling stage, the MDA content of 103 nmol g^–1^ FW in *spl29* leaves was more than double the 49 nmol g^–1^ FW in wild-type leaves (Fig. 9C). At the tillering stage, an MDA content of 378 nmol g^–1^ FW was generated in the leaves of *spl29* mutant plants, much higher than the 209 nmol g^–1^ FW in wild-type leaves (Fig. 9D). Increased MDA contents suggested that lipid peroxidation occurred in *spl29* plants, which provided further evidence of the ROS accumulation and membrane damage in *spl29* plants.

During oxidative stress, plants synthesize anti-oxidative enzymes, such as SOD and CAT, to remove ROS (Miller *et al.*, 2010). SOD catalyses the dismutation of O_2_^–^ to produce H_2_O_2_, and CAT is the major H_2_O_2_-scavenging enzyme. Thus SOD and CAT activities were examined to explore the ROS metabolic process in *spl29* plants. At the seedling stage, SOD activity was 8926U g^–1^ FW in *spl29* leaves, which was much higher than the 3208U g^–1^ FW in the wild-type leaves (Fig. 9E). A similar result was observed in leaves at the tillering stage, with a SOD activity of 12260U g^–1^ FW in *spl29* leaves compared with 2167U g^–1^ FW in the wild type (Fig. 9F). However, there was no obvious difference in CAT activity between *spl29* and wild-type leaves at both the seedling stage and the tillering stage (Fig. 9G–H). The increase of SOD activity suggests that the *spl29* mutant may actively respond to the O_2_^–^ accumulation and produce more H_2_O_2_, while the normal CAT activity may not be enough to scavenge the additional H_2_O_2_, leading to its accumulation in *spl29*.

### JA and ABA accumulate in the *spl29* mutant

**Fig. 10.**
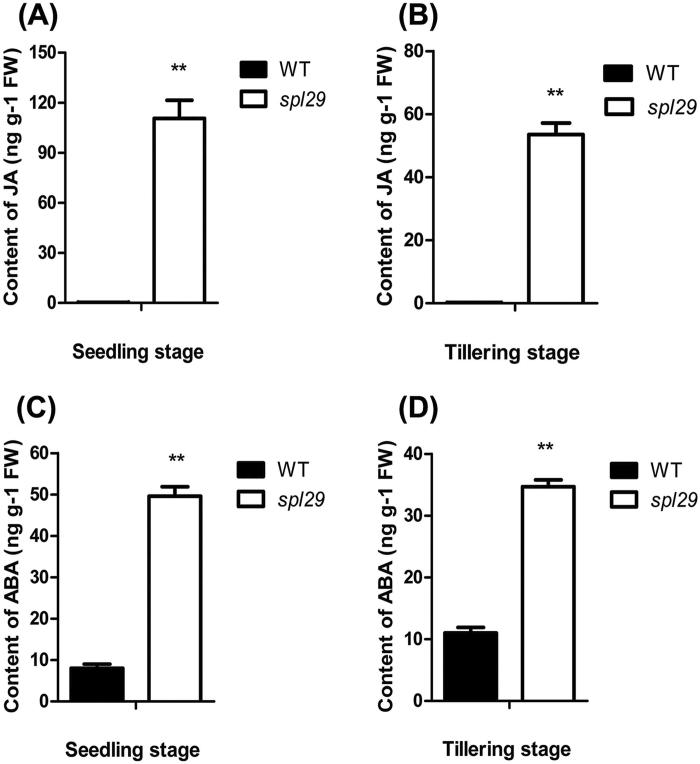
JA and ABA contents in wild-type and *spl29* leaves. (A, B) JA contents. (C, D) ABA contents. Leaf samples were analysed at the seedling and tillering stages. Data represent the mean ± SD of two biological replicates (Student’s *t*-test: **, *P* < 0.005). FW, fresh weight.

Plant hormones, such as JA and ABA, are reported to play roles in both leaf senescence and defence responses (Lim *et al.*, 2007; Robert-Seilaniantz *et al.*, 2011). This prompted us to investigate the contents of JA and ABA in *spl29*. At the seedling stage, the content of JA was 110.6ng g^–1^ FW in *spl29*, whereas JA in the wild type was hardly detectable, with a content of 0.3ng g^–1^ FW (Fig. 10A). Similar results were also found at the tillering stage, with a JA content of 53.5ng g^–1^ FW in *spl29*, but 0.2ng g^–1^ FW in the wild type (Fig. 10B). The content of ABA at the seedling stage was 49.6ng g^–1^ FW in *spl29*, compared with 8.0ng g^–1^ FW in the wild type (Fig. 10C). At the tillering stage, the content of ABA was 34.7ng g^–1^ FW in *spl29*, higher than the 11.0ng g^–1^ FW in the wild type (Fig. 10D). These results show that JA and ABA accumulate in *spl29* plants, probably playing a role in early leaf senescence and defence responses in this mutant.

## Discussion

### *SPL29* is the *UAP1* gene in rice and functional inactivation of OsUAP1 is responsible for the mutant phenotypes

From the seedling stage, the *spl29* mutant showed LM spots on every leaf. The leaf then exhibited chlorosis and soon withered (Fig. 1 and Supplementary Figure S2). It was decided that examining *spl29* might show a novel gene involved in early leaf senescence and defence responses. The *SPL29* gene was identified as LOC_Os08g10600 by map-based cloning (Fig. 2), and an NCBI BLASTP search revealed it to be a putative *UAP1* gene. Phylogenetic analysis showed that SPL29 belongs to the cluster of UAPs, which are separate from the cluster of UGPs (Fig. 3). SPL29 was confirmed as UAP1 in rice, able to perform UAP enzymatic activity (Fig. 4). The mutant phenotype of *spl29* results from its UAP1 functional inactivation (Fig. 4). UAP is an important enzyme, widely distributed in living organisms; *UAP* mutants have been discovered in *E. coli*, *S. cerevisiae*, and *D. melanogaster*, and all show abnormal development (Mengin-Lecreulx and van Heijenoort, 1993; Mio *et al.*, 1998; Schimmelpfeng *et al.*, 2006). Until now, there has been no report of *UAP* mutants in plants, nor has the *UAP* gene been identified in rice. This study identified the *UAP1* gene in rice and showed that the functional inactivation of *OsUAP1* induces early leaf senescence and defence responses.

### Early leaf senescence is induced by functional inactivation of OsUAP1

Only a few studies describe senescence as a phenotype of *spl* mutant plants, and the senescence syndrome has not been fully analysed in these mutants (Qiao *et al.*, 2010). Additionally, senescence has not been reported in UAP mutants in other species. Therefore, it was necessary to determine that early leaf senescence does indeed occur in *spl29* plants.

The major physiological change during leaf senescence is the gradual loss of chlorophyll (Jiao *et al.*, 2012; Zhang and Zhou, 2013). After the mutant phenotypes appeared, chlorophyll contents were indeed decreased in *spl29* (Fig. 5A-B). Senescence-induced *SGR* expression in *spl29* (Fig. 5C and D) may play an important role in regulating chlorophyll degradation (Park *et al.*, 2007). Reduced PSII capacity is another manifestation of leaf senescence at the physiological level (Zhang *et al.*, 2012; Panda and Sarkar, 2013). The PSII machinery in *spl29* was degraded and its function was fully depressed (Fig. 5E and F; Supplementary Figure S4). Surprisingly, *spl29* had increased capacity in each RC (ABS/RC, TR_o_/RC, ET_o_/RC, and DI_o_/RC), although this was previously seen in the senescence-induced PSII alterations of *Cucumis sativus* cotyledons (Prakash *et al.*, 2003).

The earliest and most significant change during leaf senescence at the cellular level is chloroplast breakdown (Lim *et al.*, 2007). Since the leaves initially grew normally in *spl29*, well-developed chloroplasts could be observed in many *spl29* mesophyll cells even when early leaf senescence occurred (Fig. 6B). However, chloroplasts were completely degraded in abundant dying *spl29* mesophyll cells (Fig. 6D; Supplementary Figure S5C and D), with chloroplast breakage potentially being involved in this degradation process (Fig. 6C; Supplementary Figure S5A and B). Chloroplast degradation in *spl29* is different from the autophage-mediated process described in previous reports (Jiao *et al.*, 2012), but the drastic and irreversible degradation process is nevertheless coincident with the rapid leaf senescence phenotype in *spl29*.

At the molecular level, leaf senescence is mediated by a large number of genes. The senescence-associated transcription factors *OsWRKY23*, *OsWRKY72*, and *OsNAC2* are all upregulated during leaf senescence, caused by knockdown of the H subunit gene of the glycine decarboxylase complex (Zhou *et al.*, 2013). Four *SAG*s (*Osl2*, *Osl30*, *Osl43*, and *Osl85*) are upregulated during dark-induced or natural leaf senescence (Lee *et al.*, 2001). Also the expression of photosynthesis-related genes usually decreases during leaf senescence (Lim *et al.*, 2007). Upregulation of senescence-associated transcription factors (Fig. 7A and B) and *SAG*s (Fig. 7C and D) and downregulation of photosynthesis-related genes (Fig. 7E and F) in *spl29* together support the hypothesis that early leaf senescence in this mutant is not a passive and unregulated degenerative process, but an active and well-controlled genetic programme.

The physiological, cellular, and molecular evidence all suggest that early leaf senescence happens in *spl29* plants. Functional inactivation of *OsUAP1* results in the appearance of early leaf senescence, suggesting a role for *OsUAP1* in the regulation of this process.

### Defence responses are induced by functional inactivation of OsUAP1

LMMs frequently have upregulated defence response gene expression and spontaneous appearance of HR-like lesions, both of which may contribute to enhanced resistance to pathogens (Yin *et al.*, 2000). A point mutation in *OsUAP1* results in the induction of HR-like lesions and enhanced resistance against the bacterial blight pathogen (Fig. 8A). Induced defence responses can also be reflected by the upregulation of defence response-associated genes. *PR1a* and *PBZ1* have commonly been used as molecular markers for rice defence responses (Campbell and Ronald, 2005; Tang *et al.*, 2011). Constitutively overexpressing rice *PO-C1* enhanced disease resistance and induced transcript levels of pathogenesis-related genes in transgenic carrot (Wally and Punja, 2010). Overexpression of *OsWRKY45* markedly enhanced fungal and bacterial disease resistance (Shimono *et al.*, 2012). These four defence response-associated genes (*PR1a*, *PBZ1*, *PO-C1*, and *OsWRKY45*) were all upregulated in *spl29* (Fig. 8B and C), and probably play important roles in its enhanced resistance to bacterial blight. Defence responses are induced in *spl29*, which implies a role for *OsUAP1* in disease resistance by regulating the defence response.

### ROS, JA, and ABA may play important roles in early leaf senescence and defence responses induced by OsUAP1 inactivation

ROS (O_2_^–^ and H_2_O_2_) accumulated in *spl29* (Fig. 9A and B), and the mutant also had elevated levels of MDA (Fig. 9C and D), a product of lipid peroxidation. Increased SOD activity, but normal CAT activity (Fig. 9E–H), may result in the H_2_O_2_ accumulation in *spl29* plants. Despite increased SOD activity to remove O_2_^–^, O_2_^–^ still accumulated in the mutant, and therefore the ROS accumulation in *spl29* can probably be attributed to the overproduction of O_2_^–^. ROS may play several important roles in the leaf senescence of *spl29*. ROS-mediated chloroplast degradation happens during leaf senescence (Khanna-Chopra, 2012), while lipid peroxidation by ROS has been proposed as the major cause of membrane deterioration in plant senescence (Pauls and Thompson, 1980). Thus, ROS accumulation is the most likely reason for the lipid peroxidation (Fig. 9C and D) and chloroplast membrane breakage (Fig. 6C; Supplementary Figure S5A and B) in *spl29* leaves. The onset of plant senescence can be promoted by ROS (Navabpour *et al.*, 2003). Expression patterns of the senescence-related transcription factors *OsWRKY23*, *OsWRKY72*, and *OsNAC2*, as well as the *SAG Osl85*, were all upregulated after H_2_O_2_ treatment (Zhou *et al.*, 2013). Upregulation of these four genes was also found in *spl29* plants (Fig. 7A–D). ROS probably also play a key role in the defence responses of *spl29.* The oxidative burst, a rapid and transient production of huge amounts of ROS, is one of the earliest observable aspects of a plant’s defence strategy (Wojtaszek, 1997). ROS have been implicated not only in anti-pathogen roles, but also in cellular signalling associated with the induction of defence gene expression (Desikan *et al.*, 2000). In *spl29*, the bacterial blight resistance was enhanced (Fig. 8A) and defence response-associated genes were upregulated (Fig. 8B and C).

In addition, JA and ABA levels were significantly elevated in *spl29* plants (Fig. 10). The JA and ABA signalling pathways are widely considered to play a role in modulating leaf senescence and defence responses (Lim *et al.*, 2007; Robert-Seilaniantz *et al.*, 2011; Zhang and Zhou, 2013). The accumulated JA and ABA in *spl29* (Fig. 10) are probably involved in its early leaf senescence and defence responses.

In conclusion, functional inactivation of *OsUAP1* induces early leaf senescence and defence responses in *spl29*. ROS, JA, and ABA are all likely to be involved in these two biological processes or pathways in the mutant. It is suggested that *OsUAP1* may play an important role in regulating leaf senescence and defence responses in rice.

## Supplementary material

Supplementary data can be found at *JXB* online.

Supplementary Table S1. List of PCR-based molecular markers used for map-based cloning of *SPL29*.

Supplementary Table S2. Primers for the detection of the mutation site, construction of the functionally complementary vector, and confirmation of positive transgenic plants.

Supplementary Table S3. All primers used for qRT-PCR analysis.

Supplementary Table S4. Formulae and glossary of terms used in the analysis of chlorophyll *a* fluorescence transients.

Supplementary Table S5. Segregation of F_2_ populations from three crosses.

Supplementary Table S6. Sequencing of the mutation site in different rice cultivars and F_2_ plants.

Supplementary Figure S1. Determination of reference genes for normalisation in qRT-PCR analysis.

Supplementary Figure S2. Phenotype of the second-emerged leaf after germination in wild-type and *spl29* plants.

Supplementary Figure S3. SDS-PAGE of proteins.

Supplementary Figure S4. Different expression of relative variable fluorescence.

Supplementary Figure S5. Ultrastructure in mesophyll cells of *spl29*.

## Funding

This work was supported by the Key Grant Project of the Chinese Ministry of Education (Grant No. 313039), Specialized Research Fund for the Doctoral Programme of Higher Education (20130141110069), National Programme of Transgenic Variety Development of China (Grant Nos 2011ZX08001-001 and 2011ZX08001-004), and the National Natural Science Foundation of China (91017013, 91217309, 20825520, and 31200208).

## Supplementary Material

Supplementary Data

## Abbreviations:

LMMs: lesion mimic mutants
UAP: UDP-*N*-acetylglucosamine pyrophosphorylase
GlcNAc-1-P: *N*-acetylglucosamine-1-phosphate
GalNAc-1-P: *N*-acetylgalactosamine-1-phosphate
UDP-GlcNAc: UDP-*N*-acetylglucosamine
UDP-GalNAc: UDP-*N*-acetylglucosamine
spl29: *spotted leaf 29*
ZH11: Zhonghua 11
LM: lesion mimic
RGAP: Rice Genome Annotation Project
GST: glutathione *S*-transferase
CDS: coding sequence
NMR: nuclear magnetic resonance
qRT-PCR: quantitative, real-time reverse transcription polymerase chain reaction
FW: fresh weight
ROS: reactive oxygen species
MDA: malondialdehyde
SOD: superoxide dismutase
CAT: catalase
JA: jasmonic acid
ABA: abscisic acid
ORF: open-reading frame
NLM: non-lesion mimic
UGP: UDP-glucose pyrophosphorylase
SGR: *STAY GREEN*
PSII: photosystem II
SAGs: senescence-associated genes
HR: hypersensitive response
NBT: nitro blue tetrazolium
DAB: 3,3’-diaminobenzidine.
