# Supplementary material for: Functional inactivation of UDP-N-acetylglucosamine pyrophosphorylase 1 (UAP1) induces early leaf senescence and defence responses in rice
Source: J Exp Bot. 2014 Nov 15;66(3):973–87. doi: 10.1093/jxb/eru456 (PMC4321554; doi:10.1093/jxb/eru456)
Supplement: Supplementary Data [file supp_66_3_973__index.html]

Functional inactivation of UDP-N-acetylglucosamine pyrophosphorylase 1 (UAP1) induces early leaf senescence and defence responses in rice — Functional inactivation of UDP-N-acetylglucosamine pyrophosphorylase 1 (UAP1) induces early leaf senescence and defence responses in rice — Supplementary Data 

# Functional inactivation of UDP-*N*-acetylglucosamine pyrophosphorylase 1 (UAP1) induces early leaf senescence and defence responses in rice

## Supplementary Data

Data files

**Files in this Data Supplement:**

- Supplementary Data - Supplementary Data
