# Supplementary material for: Functional inactivation of UDP-N-acetylglucosamine pyrophosphorylase 1 (UAP1) induces early leaf senescence and defence responses in rice
Source: J Exp Bot. 2014 Nov 15;66(3):973–87. doi: 10.1093/jxb/eru456 (PMC4321554; doi:10.1093/jxb/eru456)

Functional inactivation of UDP-*N*-acetylglucosamine pyrophosphorylase 1 (UAP1) induces early leaf senescence and defence responses in rice (*Oryza sativa*)

Zhaohai Wang[1], Ya Wang[1], Xiao Hong[1], Daoheng Hu[1], Caixiang Liu[2], Jing Yang[1], Yang Li[1], Yunqing Huang[3], Yuqi Feng[3], Hanyu Gong[1], Yang Li[1], Gen Fang[1], Huiru Tang[2,4], Yangsheng Li[1]*

Supplementary Data

Table S1 List of PCR-based molecular markers used for map-based cloning of *SPL29*.

Table S2 Primers for detection of mutation site, construction of functional complementary vector and confirmation of positive transgenic plants.

Table S3 All primers used for qRT-PCR analysis.

Table S4 Formulae and glossary of terms used in the analysis of chlorophyll a fluorescence transient.

Table S5 Segregation of F_2 populations from three crosses.

Table S6 Base sequencing of the mutation site in different rice cultivars and F_2 plants.

Fig. S1 Determining reference genes for normalisation in qRT-PCR analysis.

Fig. S2 Phenotype of the second-emerged leaf after germination in wild-type and *spl29* plants.

Fig. S3 SDS/PAGE of proteins.

Fig. S4 The different expressions of relative variable fluorescence.

Fig. S5 Ultrastructure in mesophyll cells of *spl29*.

Table S1 List of PCR-based molecular markers used for map-based cloning of *SPL29*

Marker name	Forward primer	Reverse primer
M0373 (Chr08MM0373)	ACCTCGTCTTCCTCCTCCAT	AGCTCGATAGCTAGGTCGCA
M0851 (Chr08MM0851)	ATGGGACGGAGGGAGTAGTT	CGGTGCTCTGCTTCTTTTTC
M0979 (Chr08MM0979)	TTTCTCTATGCGTGTGCTGC	AATTGCTGGGGAGGATTTCT
M1037 (Chr08MM1037)	TCAGCACTCACAGAAGTCTAATCACG	GTCGTTGATCGATCGAGTAGTGG
M1077 (Chr08MM1077)	CAAAGCAAATGCTCACGAAA	AATGGAGGCTAACGAACACG
M1230 (Chr08MM1230)	TGCATCGGACGAGTATCCTT	GACAAAAGTTCCGGCTTGAG
M1412 (Chr08MM1412)	CAGATTATTCAGACACACCAGACG	GGAATAGGGTATCTCATCTCTCTTGC
S1	TTCAACAATCAACATGCAAT	TCATTGCTGAACCTGTGAT
S6	GCGATGGGATCTCTACAAC	AATTAGTTGCTTTCTGGTGG
S8	GTCAGGAAGCAAATTTTCAG	TTTTGAAAGTTTGAAGGGAA
S15	TACTGAGCTAACTGGGTCGT	TTAATCACTTACACCCGAGC
S19	CCATCCAAAAATATATGTCAAA	ATGAATGGTTGCCTCTACAT
S26	AGTTTTGTGAAATTTACTCTACTGA	CTACCAACAACCCATTTCTC
S32	CATGCAAACAAAAATAACCA	TTGTACAAACTGTCAACCCA
S33	GCTTAATACGCAGAGCCATA	AGGCAGTCAAAAAGGTAAAA
S40	AAAAATCTACTGTTGGGGCT	TTTTCTCAGGGAAGTGGTTA

Table S2 Primers for detection of mutation site, construction of functional complementary vector and confirmation of positive transgenic plants

Primer name	Forward primer	Reverse primer
*SPL29*Mutation	CCCATTCTTCCATCATTCCG	CATATTTCACACCTCTTCCAGCC
*SPL29*Pro	***caqtGCTCTTCatag***cgtttgaccagatgtcggaa	***cagtGCTCTTCa***aggagagaggctgctgctgc
*SPL29*Gene	***cagtGCTCTTCacct***actaataatctccccaagat	***cagtGCTCTTCa***ttgaaaaaggaaaatgtgta
*SPL29*Ter	***cagtGCTCTTCacaa***gtttgcaatacaattaatgc	***cagtGCTCTTCagac***gttctacagcggttcagtgt
Bar178	AGAAACCCACGTCATGCCAGT	ACGCTCTACACCCACCTGCT
GST-SPL29/*spl29* CDS	***cgGGATCC***ATGGCGGAGATCGTGGTG	***cgGAATTC***CTAAAATGAAATCTCACTCGGTGC

Note: In primers of *SPL29*Pro, *SPL29*Gene and *SPL29*Ter, underlined sequences for are used for digesting-link one step reaction, and the upper letters are the Sap I recognizing site. In primers of *GST-SPL29*/*spl29* CDS, the restriction enzyme sites BamH I and EcoR I were introduced in the forward and reverse primer, respectively.

Table S3 All primers used for qRT-PCR analysis

Primer name	Forward primer	Reverse primer	Amplicon Length (bp)	Annealing (℃)	PCR efficiencey(%)	R^2
SGR	GCAATGTCGCCAAATGACG	GCTCACCACACTCATTCCCTAAAG	132	60	94.4	0.995
OsWRKY23	TCCAGTTCCTCTCCCAGTTCTAA	CACATTGTTCTCCTTTTCTTCCC	186	60	86.5	0.997
OsWRKY72	CACCACAAATCACATCTACTCCG	GCTGAAGGGAAGAGAGGTGAG	81	60	92.5	0.997
OsNAC2	AAAAACAACCGCATTGGCAG	AGTCCTCATCTCCTCTGTCTAATCC	166	60	85.9	0.998
Osl2	GCAGACAACAAATCGCCAAAT	TCTCCAGCAACTCTAACCAGCAT	107	60	97.7	0.998
Osl30	AACCTTTTTCTTGGAGATGATACAA	CTTGAACTGTAGGGGCTTGCTT	176	60	91.2	0.999
Osl43	TGTGACAAGTGCTAATAATACATACGA	CCAGACCTTCCAAAGAATCCAAC	108	60	93.0	0.999
Osl85	TCCAGGATGTGATGAGGATTATTC	GCGTGCTGTAGTTCAGTCTGTAAAG	121	60	96.7	0.998
rbcS	TACCTGCTCCGTTCCAAGTG	CGGGGGATCTGTGGTTCTC	82	60	95.4	0.998
lhcA	GTCTGTGGTTTGACCCGCT	GAGCCGCCCGTTCTTGA	89	60	97.1	0.999
lhcB	GCTCAAGGTGAAGGAGATCAAGA	ATGCGTTGTTGTTGACGGG	140	60	103.4	0.998
rbcL	CTACTTCTTCACATTCACCGAGC	GCGTTCCCCTTCTAACTTACCTA	153	60	89.0	1
psaA	GCGAGCAAATAAAACACCTTTC	GTACCAGCTTAACGTGGGGAG	164	60	96.0	0.998
psbA	TGACCAATAGGGTAAATCAAGAAAA	TGTTATATGGGTCGTGAGTGGG	122	60	86.5	0.999
petD	TAATGGTTTCTGTGCCGACG	CCTAAAGTTAAGGATTTTTCAATGGG	175	60	96.9	0.996
ndhA	CAAAGCGATATCCCAAAGGAAT	CCGCATCGATACAACAACGTAT	204	60	91.1	0.999
atpA	GATCTCTCCAAACAGGCACAAG	CGGCTCTTTCTAAAAGGCGTG	121	60	103.3	0.999
PR1a	TGCATGTATGGACATGTAGTGTCATA	TACACTAAGCAAATACGGCTGACA	133	60	88.6	0.998
PBZ1	CGCAAGTCATGTCCTAAAGTCG	ATGCCATAGTAGCCATCCACG	201	60	93.1	0.995
PO-C1	TCGGACCAGGTGCTCTTCAA	CTGATCTGGCCGTTTGTTCC	149	60	92.3	0.996
OsWRKY45	GAAGAATCATGGATGGACACGG	GACACATCAACAAGGAATTTACAAAC	166	60	85.8	0.999
TI	CGACATCATCAACTCCGCCAC	CCTCTTCAGACATCTTCCCACG	83	60	97.4	0.997

ARF	ATGAAAGGAAGACATGGCGG	TGGTGGTGGAACCTAAAGAGC	126	60	95.2	0.999
EF-1α	GCTGCTGCAACAAGATGGATG	CAGAGATGGGAACGAAGGGAA	135	60	90.8	0.999
UBC	GTGCAGCGAGAAAAGTCAGC	GAACTTGCGGAGGAAGGAGAG	172	60	95	0.998
Profilin-2	CCAACTGGTCTTTTCCTTGGG	GGGGTCATCGGCTCATCATAG	152	60	93.9	0.999
Actin1	GGAAGTACAGTGTCTGGATTGGAG	TCTTGGCTTAGCATTCTTGGGT	155	60	98.1	0.995

Table S4 Formulae and glossary of terms used in the analysis of chlorophyll a fluorescence transient

Fluorescence parameters	Description
Extracted parameters	
F_t	Fluorescence intensity at time t after onset of actinic illumination
$F_{20\ \mu s}$	Minimum reliable recorded fluorescence at 20 µs with the Handy-PEA-fluorimeter
$F_{100\ \mu s}$ and $F_{300\ \mu s}$	Fluorescence intensity at 100 and 300 µs, respectively
F_J and F_I	Fluorescence intensity at the J-step (2 ms) and the I-step (30 ms),respectively
$F_o \approx F_{20\ \mu s}$	Maximum recorded (= minimum possible) fluorescence at O-step when all PSII RCs are open
$F_m = F_P$	Maximum recorded (= maximum possible) fluorescence at P-step when all PSII RCs are closed
Area	Total complementary area between fluorescence induction curve and $F = F_m$
Derived parameters	
Selected OJIP parameters	
$V_J = (F_{2\ ms} - F_o)/(F_m - F_o)$	Relative variable fluorescence at the J-step (2 ms)
$V_I = (F_{30\ ms} - F_o)/(F_m - F_o)$	Relative variable fluorescence at the I-step (30 ms)
$M_o = 4\ (F_{300\ \mu s} - F_o)/(F_m - F_o)$	Approximated initial slope (in ms^{-1}) of the fluorescence transient V = f(t)
$S_m = EC_o/RC = Area/(F_m - F_o)$	Normalized total complementary area above the OJIP (reflecting multipleturnover QA reduction events) or total electron carriers per RC
Yields or flux ratios	
$\varphi_{Po} = TR_o/ABS = 1-(F_o/F_m) = F_v/F_m$	Maximum quantum yield of primary photochemistry at t = 0
$\varphi_{Eo} = ET_o/ABS = (F_v/F_m) \times (1 - V_J)$	Quantum yield for electron transport at t = 0
$\psi_{Eo} = ET_o/TR_o = 1-V_J$	Probability (at time 0) that a trapped exciton moves an electron into the electron transport chain beyond QA^-
$\delta_{Ro} = RE_o/ET_o = (1 - V_I)/(1 - V_J)$	Efficiency with which an electron can move from the reduced intersystem electron acceptors to the PSI end electron acceptors
Phenomenological fluxes or activities expressed per excited cross section (CS)	

$ABS/CS_o \approx F_o$	Absorpted energy flux per CS at t = 0
$TR_o/CS_o = (ABS/CS_o) \times \varphi_{Po}$	Trapped energy flux per CS at t = 0
$ET_o/CS_o = (ABS/CS_o) \times \varphi_{Eo}$	Electron transport flux per CS at t = 0
$DI_o/CS_o = (ABS/CS_o) - (TR_o/CS_o)$	Dissipated energy flux per CS at t = 0
$ABS/CS_m \approx F_m$	Absorpted energy flux per CS at $t = t_{Fm}$
$TRo/CS_m = (ABS/CS_m) \times \varphi_{Po}$	Trapped energy flux per CS at $t = t_{Fm}$
$ET_o/CS_m = (ABS/CS_m) \times \varphi_{Eo}$	Electron transport flux per CS at $t = t_{Fm}$
$DI_o/CS_m = (ABS/CS_m) - (TR_o/CS_m)$	Dissipated energy flux per CS at $t = t_{Fm}$
Density of reaction centers (RCs)	
$RC/CS_o = \varphi_{Po} \times (ABS/CS_o) \times (V_J/M_o)$	Amount of active PSII RCs per CS at t = 0
$RC/CS_m = \varphi_{Po} \times (ABS/CS_m) \times (V_J/M_o)$	Amount of active PSII RCs per CS at $t = t_{Fm}$
Specific fluxes or activities expressed per reaction center (RC)	
$ABS/RC = M_o/V_J/\varphi_{Po}$	Absorpted energy flux per RC at t = 0
$TR_o/RC = M_o/V_J$	Trapped energy flux per RC at t = 0
$ET_o/RC = (M_o/V_J) \times \psi_{Eo} = (M_o/V_J) \times (1-V_J)$	Electron transport flux per RC at t = 0
$DI_o/RC = (ABS/RC) - (TR_o/RC)$	Dissipated energy flux per RC at t = 0
Performance index	
$PI_{ABS} = (RC/ABS) \times (\varphi_{Po}/(1 - \varphi_{Po})) \times (\psi_{Eo} /(1 - \psi_{Eo}))$	Performance index (PI) on absorption basis
$PI_{CSo} = (RC/CS_o) \times (\varphi_{Po}/(1 - \varphi_{Po})) \times (\psi_{Eo} /(1 - \psi_{Eo}))$	Performance index (PI) on CS basis at t = 0
$PI_{CSm} = (RC/CS_m) \times (\varphi_{Po}/(1 - \varphi_{Po})) \times (\psi_{Eo} /(1 - \psi_{Eo}))$	Performance index (PI) on CS basis at $t = t_{Fm}$
$PI_{tot, ABS} = (RC/ABS) \times (\varphi_{Po}/(1-\varphi_{Po})) \times (\psi_{Eo}/(1 - \psi_{Eo})) \times (\delta_{Ro}/(1 - \delta_{Ro}))$	Total PI, measuring the performance up to the PSI end electron acceptors on absorption basis
$PI_{tot,CSo} = (RC/CSo) \times (\varphi_{Po}/(1-\varphi_{Po})) \times (\psi_{Eo}/(1 - \psi_{Eo})) \times (\delta_{Ro}/(1 - \delta_{Ro}))$	Total PI, measuring the performance up to the PSI end electron acceptors on CS basis at t = 0
$PI_{tot,CSm} = (RC/CSM) \times (\varphi_{Po}/(1-\varphi_{Po})) \times (\psi_{Eo}/(1 - \psi_{Eo})) \times (\delta_{Ro}/(1 - \delta_{Ro}))$	Total PI, measuring the performance up to the PSI end electron acceptors on CS basis at $t = t_{Fm}$

Table S5 Segregation of F_2 populations from three crosses

Cross	Wild type	LM mutant	Total	χ^2(3:1)	*P* value
Guangzhan63s/*spl29*	1136	365	1501	0.338	0.84
10N056/*spl29*	305	111	416	0.542	0.76
Yuehui9113/*spl29*	278	95	373	0.022	0.99

Note：The female/male parents are showed in the cross. $\chi^2 < \chi^2_{0.05}$ =3.84 is for 3:1 segregation ratio. *P*>0.05 is considered as significant.

Table S6 Base sequencing of the mutation site in different rice cultivars and F_2 plants

Rice cultivars		F_2 NLM plants		F_2 LM plants	
spl29	T	F_2NLM-1	G	F_2LM-1	T
ZH11	G	F_2NLM-2	G/T	F_2LM-2	T
Kongyu131	G	F_2NLM-3	G	F_2LM-3	T
YTB	G	F_2NLM-4	G/T	F_2LM-4	T
MP3	G	F_2NLM-5	G/T	F_2LM-5	T
02428	G	F_2NLM-6	G	F_2LM-6	T
Chuanxiang29B	G	F_2NLM-7	G/T	F_2LM-7	T
Songjin13	G	F_2NLM-8	G/T	F_2LM-8	T
Songhei05-277	G	F_2NLM-9	G/T	F_2LM-9	T
Niponbare	G	F_2NLM-10	G	F_2LM-10	T
Guangzhan63s	G	F_2NLM-11	G/T	F_2LM-11	T

Note: F_2 plants were randomly selected from F_2 population of Guangzhan63s and *spl29*. NLM: non-lesion-mimic; LM: lesion-mimic.

Fig. S1 Determining reference genes for normalisation in qRT-PCR analysis. (A) Ranking six reference genes to recommend multiple reference genes by geNorm to normalise gene expression in leaf samples. *Arrow* indicates the best combination of multiple reference genes. (B) Determination of the optimal number of reference genes for accurate normalisation. *Arrow* indicates the optimal number.

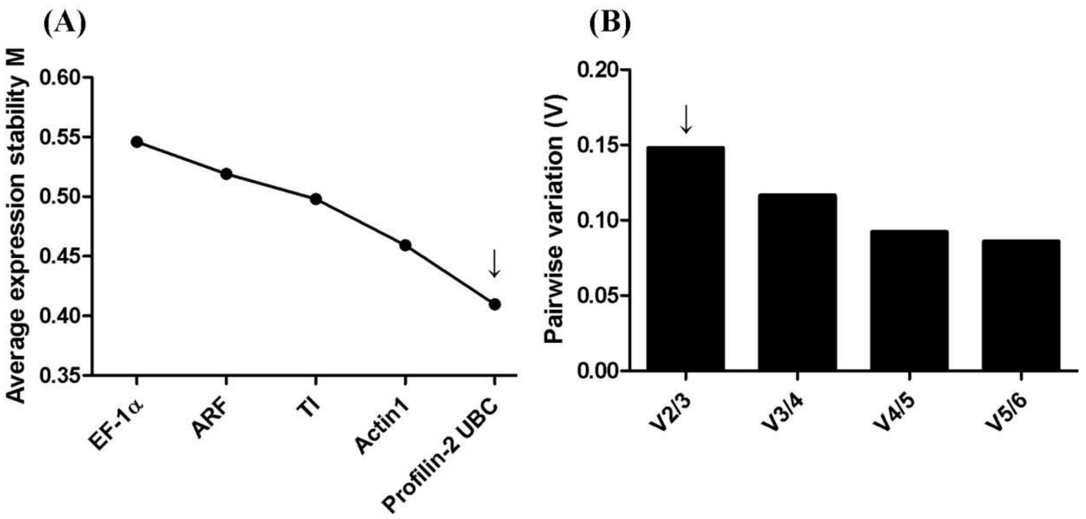

Fig. S2 Phenotype of the second-emerged leaf after germination in wild-type and *spl29* plants.

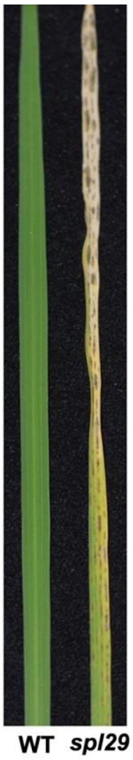

Fig. S3 SDS/PAGE of proteins. Lane 1, prestained protein ladder. Total soluble proteins from *E.coli* cells expressing control empty vector (lane 2), recombinant SPL29 (lane 3) and recombinant spl29 (lane 4). Purified GST (lane 5), GST-SPL29 (lane 6) and GST-spl29 (lane 7). Bands of GST, GST-SPL29 and GST-spl29 are indicated by *arrows*, respectively.

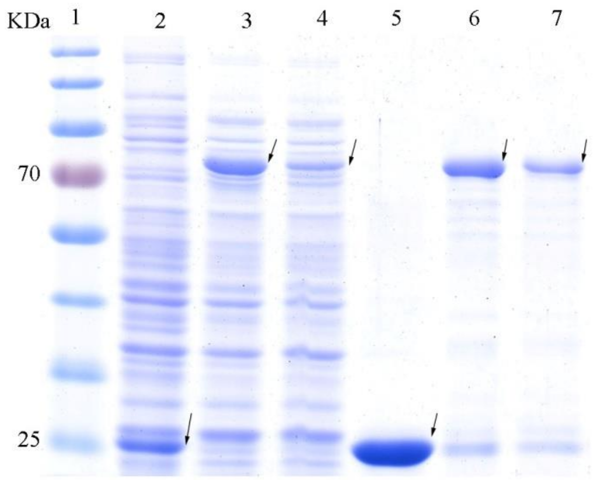

Fig. S4 The different expressions of relative variable fluorescence: between F_o and F_J: (A) $\Delta W = W_{k(spl29)} - W_{k(WT)}$, $W_k = (F_t - F_o)/(F_J - F_o)$; between F_o and F_m: (B) $\Delta V_t = V_{t(spl29)} - V_{t(WT)}$, $V_t = (F_t - F_o)/(F_m - F_o)$; between F_o and $F_{300\mu s}$: (C) $\Delta L_{band} = L_{band(spl29)} - L_{band(WT)}$, $L_{band} = (F_t - F_o)/(F_{300\mu s} - F_o)$; (D) IP phase: $(F_t - F_I)/(F_I - F_o)$.

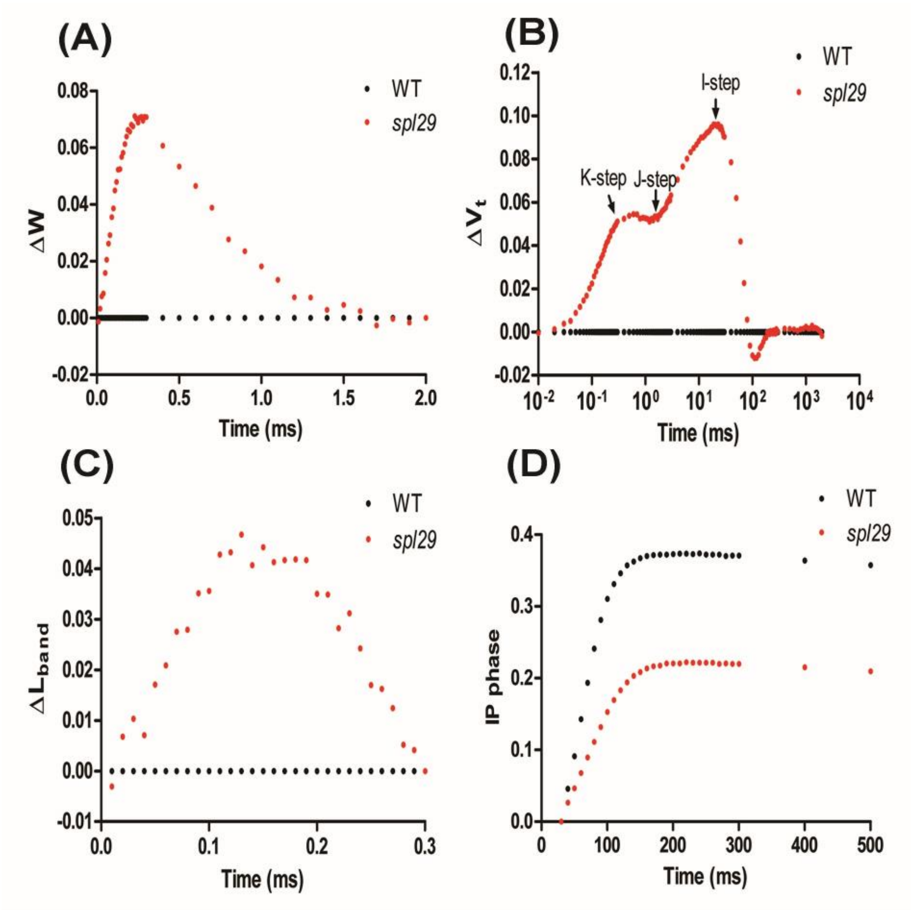

Fig. S5 Ultrastructure in mesophyll cells of *spl29*. (A-B) Chloroplast breakage. (C-D) Chloroplast was rarely seen in the dying mesophyll cells. C, chloroplast; CE, chloroplast envelope; M, mitochondrion. All scale bars represent 1μm.

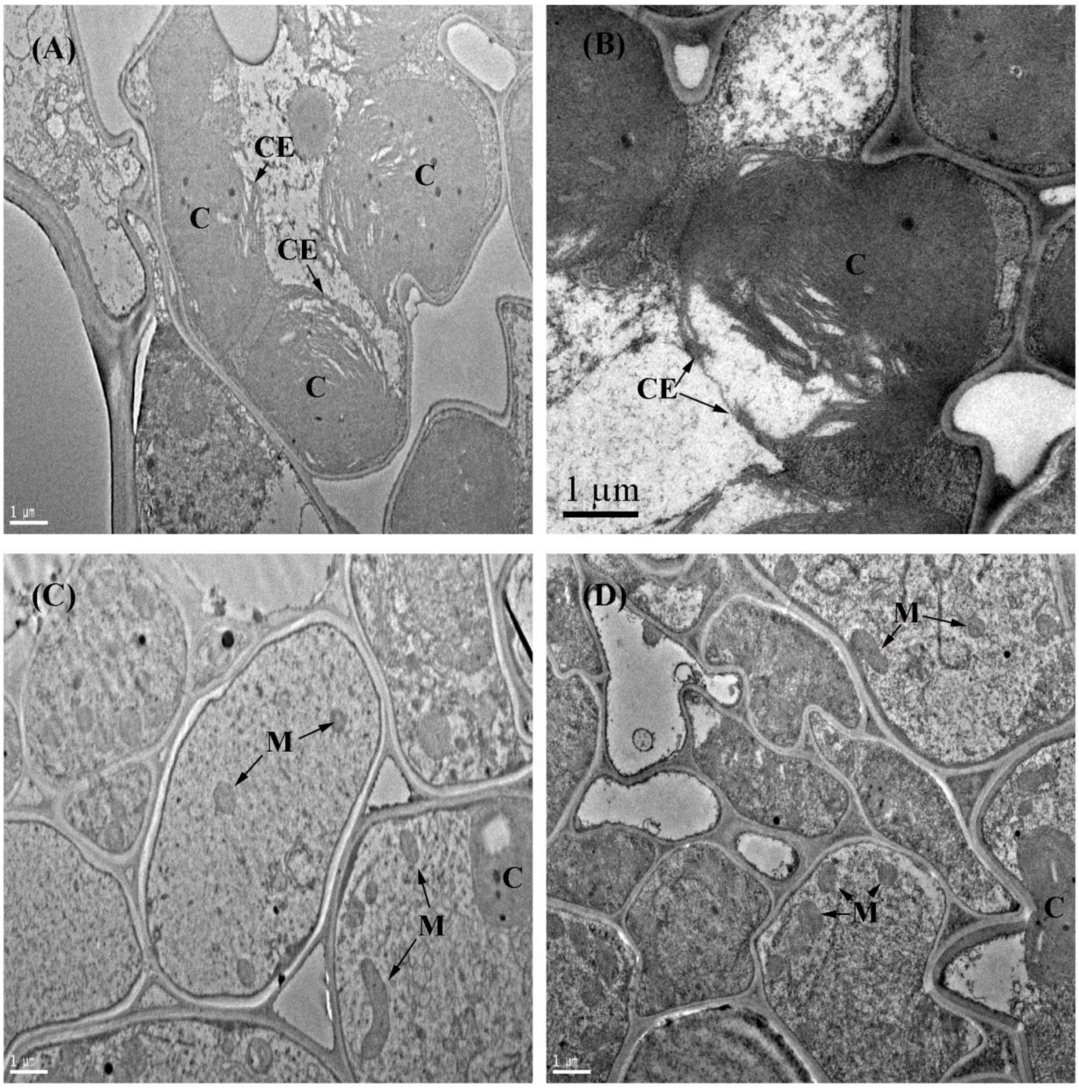

Supplement: Supplementary Data [file supp_eru456_jexbot126664_file001.pdf]
